# A multinational cross-sectional survey on the use of AI-based voice assistance systems in emergency medical services

**DOI:** 10.1186/s13049-026-01695-1

**Published:** 2026-09-15

**Authors:** Clemens Möllenhoff, Oddvar Uleberg, Matthias Bender, Thomas Neumuth, Max Rockstroh

**Affiliations:** 1 Innovation Center Computer Assisted Surgery (ICCAS), Leipzig University, Semmelweisstraße 14, 04103 Leipzig, Germany; 2 Department of Circulation and Medical Imaging, Norwegian University of Science and Technology (NTNU), Trondheim, 7006 Norway; 3 Department of Emergency Medicine and Pre-Hospital Services, St. Olav’s University Hospital, Trondheim, 7006 Norway; 4 Department of Research and Development, Norwegian Air Ambulance Foundation, Oslo, 0103 Norway; 5 Engineering and Information Technology, Institute of Medical Informatics (I4MI), Bern University of Applied Sciences, Biel, 2502 Switzerland

**Keywords:** Artificial intelligence (AI), Digital technologies, Emergency medical services (EMS), Emergency medicine, Human–computer interaction (HCI), Natural language processing (NLP), Prehospital care, User study, Voice assistant, Workflow integration

## Abstract

**Background:**

Emergency medical services (EMS) worldwide face increasing demands that require improvements in efficiency and quality of care. Voice assistance systems (VAS) based on Artificial Intelligence (AI) can support staff in prehospital settings through contactless, intuitive operation. However, evidence regarding their application in EMS is limited but desired. To date, no large-scale study has systematically explored potential functions, user acceptance, or implementation challenges of voice assistance systems in prehospital emergency medicine. This study aimed to explore the possibilities and challenges of designing and implementing a VAS tailored for EMS from a user perspective. Emergency medical personnel from three European countries were invited to share their requirements, expectations, and anticipated obstacles across different systems and countries.

**Methods:**

An exploratory, cross-sectional online survey was conducted in Germany, Norway, and Switzerland during the summer of 2024 using a 34-item self-administered questionnaire. Eligible participants were EMS professionals, including physicians, paramedics, emergency medical technicians, and students. Collected data were analyzed using descriptive statistics and a large language model. The reporting of this study adhered to the CROSS checklist.

**Results:**

A total of 587 responses were received, of which 186 (32%) were excluded, leaving 401 responses for final analysis. Participants reported occasional use of AI applications and voice assistants in personal or work settings and demonstrated a high level of technical proficiency. In EMS, several digital tools are in use today, mainly concerning documentation, knowledge access, hospital pre-notification and occupancy checks. Most respondents reported a rather positive attitude towards the use of voice assistants during missions, expecting reduced workload and improved quality of care; however, none were aware of an EMS-specific voice assistant to date. Desired features included language translation, patient history summarization, hospital pre-notification, medical information retrieval, and case documentation. Key requirements for adoption were reliability, usability, and fast system response times, while major implementation challenges involved staff acceptance, funding, and data protection.

**Conclusion:**

Developing AI-based voice assistants with emergency medical service–specific functionalities is desirable and likely to be well accepted if user requirements are addressed. Future steps include prototype-based field testing and evaluating measurable improvements in care delivery.

**Clinical trial number:**

Not applicable.

**Supplementary Information:**

The online version contains supplementary material available at https://doi.org/10.1186/s13049-026-01695-1.

## Background

Emergency Medical Services (EMS) in many European countries are facing growing challenges. Primarily, the demographic transition with an aging population leads to increased patient volumes, while workforce numbers decline [1]. Furthermore, employee retention in EMS is decreasing. For instance, only 44% of current paramedic apprentices in Germany and 50% in Switzerland plan to remain in their profession for more than 10 years [2, 3]. Meanwhile, emergency call volumes continue to rise, as illustrated by a 10% increase in Germany between 2020 and 2023 [4]. Comparable increases have been observed in Switzerland (+ 20% over six years) and Norway (+ 20% ambulance utilization and + 63% acute responses over the past 12 years) [5, 6]. In addition, healthcare systems are becoming increasingly complex, with care not only delivered in hospitals but also through local clinics, mobile units and remote services [7, 8]. Therefore, this complexity can make it difficult for patients to navigate care pathways, leading to increased EMS demand when emergency numbers are called because of uncertainty.

To ensure future resilience, EMS providers should maximize resource efficiency and improve operational quality. Innovative technologies could represent one building block in addressing these goals. Artificial intelligence (AI) is of particular interest at present. This term refers to “systems that display intelligent behavior by analyzing their environment and taking actions – with some degree of autonomy – to achieve specific goals” [9]. An AI system “infers, from the input it receives, how to generate outputs such as predictions, content, recommendations, or decisions” [10].

Several AI applications in prehospital emergency care at different stages of practical maturity can be identified in the literature: At the dispatch level, AI-based large language models (LLM) like ChatGPT have been proposed to support triage by transcribing emergency calls, extracting key information, prioritizing cases during peak demand, and enabling real-time translation for non-native speakers [11]. Artificial Intelligence has also shown promise in early recognition of critical conditions during phone calls [12]. During patient care, AI can continuously analyze vital signs obtained via ambulance-brought medical devices or patient-owned wearables, detect deterioration, trigger smart alarming, and support diagnostic reasoning [13]. Additionally, AI may enhance telemedical consultations by transcribing communication, provide access to clinical guidelines, and supporting triage and hospital assignment, factoring in vital data, traffic, weather, and hospital capacity [11]. However, none of the reported applications has been implemented in daily routine or undergone continuous real-world validation to date.

To enable future translation of such use cases into routine care, Norway has developed its own strategy for the use of AI in healthcare [14]. Also, the recent 2023 proposal paper to reform emergency medical service and acute care in Germany recommends integrating AI applications into ambulances but specifies only few concrete use cases [15]. The Swiss strategy paper on health policy development towards 2030 recognizes AI as a disruptive innovation that is to be continuously integrated into healthcare provision [16].

Despite the general potential of AI in EMS as presented in the literature, a particular sub-technology remains underexplored: Voice commands as a Human-Machine-Interface (HMI) to interact with these intelligent systems. Yet, voice interaction is particularly well-suited to EMS environments due to high mobility, hygiene constraints, stress, and the need for visual focus on patients and situations. Advances in automatic speech recognition (ASR) now enable accurate transcription with low error rates and allow the extraction and processing of relevant semantic information, even in noisy environments [17, 18]. Hence, AI-based voice assistance systems (VAS), as conceptually known from consumer applications like Apple Siri, Amazon Alexa or Google Assistant, represent a promising solution for EMS use.

In this study, voice assistance systems for Emergency Medical Services (EMS-VAS) were defined as mobile knowledge-driven, single-point-of-contact agent systems for EMS personnel, providing end-to-end functionalities to assist during emergency response. Drawing on the integration of multiple AI technologies, they facilitate natural language interaction, access to structured knowledge repositories, third-party communication via dedicated interfaces, and output through speech synthesis or graphical interfaces.

Most EMS-VAS presented to date focus on two isolated applications: First, Decision Support Systems continuously record conversations between patients and emergency personnel, extract relevant data, and suggest appropriate treatment and interventions [19–22]. Second, other systems aim to capture and document the entire emergency treatment process by converting recorded conversations into comprehensive medical case reports that can be shared with emergency room (ER) staff [23–26].

Although the first speech-based documentation system in EMS was proposed in 1999 [27], research and real-world implementations of such systems in prehospital settings remain limited, with only a small number of studies reported in the literature. Current solutions, whether intended for decision support, documentation, or both, remain prototypes that have only been evaluated in laboratory environments [19, 21–23] or on retrospective datasets [20, 24, 26]. Evidence from prospective real-world deployments remains largely absent, and the generalizability of such systems across EMS organizations, protocols, languages, and operational contexts has yet to be demonstrated [26, 28]. A scoping review on voice-based documentation in EMS conducted in 2024 identified only eight relevant publications since 2014 [28]. While interest in this field is growing, a research gap remains: no large-scale studies have yet explored user requirements and opinions [28]. However, when conceptualizing and developing AI-based VAS for EMS, it is essential to incorporate the perspective of future end users, namely paramedics, nurses, and physicians. Early integration of their feedback is key to ensuring successful implementation into practice.

The aim of this study was to identify (1) the digital systems currently in use in EMS as the current state-of-the-art, (2) the functional and non-functional requirements desired for a specialized voice assistant, (3) the anticipated challenges associated with its implementation, and (4) the level of general user acceptance that can be expected. The primary focus was put on the perspectives of EMS professionals as eventual end users. By including participants from three different countries, the study aimed to examine the research question in the context of different structural and organizational backgrounds and to highlight regional differences.

## Methods

This user study was conducted as an exploratory, cross-sectional online survey among prehospital emergency personnel in Germany, Norway, and Switzerland. Data were collected during summer 2024 using a 34-item, self-administered questionnaire. The reporting of this study followed the Consensus-based Checklist for Reporting of Survey Studies (CROSS) guidelines [29].

### Study setting

All three participating countries operate mature European EMS systems with comparable professional standards, above-average OECD funding, multi-tiered qualification pathways, established interprofessional collaboration, and largely standardized equipment [30–36]. This comparability allows for a meaningful comparison of opinions among medical staff across these systems. At the same time, differences in their organizational structures, funding structure, workforce characteristics, mission volumes, and geographic contexts may provide different perspectives on the given research questions. Detailed characteristics are presented in Table 1.

**Table 1:** Overview of the characteristics of the countries studied and their EMS systems (rounded values, as of 2024 or last available year) [30–36]

Category	Germany	Norway	Switzerland
EMS structure	Decentralized (nationwide frameworks, responsibility and organization on county level)	Centralized	Decentralized (cantonal level)
Funding model	Public health insurance + state support	Fully tax-based	Mandatory health insurance, private co-payments, cantonal subsidies, private donations
Annual EMS missions	14,000,000	738,000	460,000
EMS workforce (FTE)	66,000	4,800	2,500
Population	84 million	6 million	9 million
Country size (km^2^)	349,000	364,000	40,000
Population density (population per km^2^)	241	16	225
Geographic characteristics	Large urban agglomerations and rural areas; predominantly lowland, some alpine and coastal regions.	Predominantly sparsely populated, mountainous with fjords and remote regions, substantial variation in climate	Moderately populated, mountainous
EMS leadership model	Paramedic-led, frequent physician support via rendezvous system	Protocol-driven paramedics, limited physician involvement (except air ambulance)	Hybrid model, regional variation in physician/anesthesia nurse involvement
Multi-tiered qualifications	Yes	Yes (unique: bachelor’s degree in paramedicine)	Yes
EMS vehicle standards	European standard (EN1789) with local additions		

### Study design and data collection

#### Design and distribution of questionnaire

Country-specific questionnaires were developed with identical content adapted to local EMS structures and terminology, and provided in German, English, and Norwegian. The survey was created and administered via LimeSurvey (LimeSurvey GmbH, Hamburg, Germany), drawing on prior research and current literature. Translation and cultural validation were performed by native-speaking members of the author team actively working in their respective EMS systems to ensure accurate transfer of country-specific terminology. During development, the questionnaire was iteratively refined through three rounds of pilot testing with 12 eligible participants, who evaluated wording clarity, grammar, and technical functionality of the web-based form. No formal psychometric validation (e.g., reliability or construct validity testing) was applied.

The survey comprised 34 question items across seven categories (Supplemental File 1), of which 28 were analyzed for this work. This included numeric and text entries, single/multiple-choice items, five-point Likert scales, prioritization tasks, and a modified Kano-style item [37]. Unless noted otherwise, Likert scores ranged from 1 (“strongly agree”) to 5 (“strongly disagree”), with 3 representing a neutral response. The survey was accessible online for six weeks (28 July to 8 September 2024) [38]. As a starting point, professional networks facilitated access to EMS personnel to recruit participants. To further boost involvement in the user study, multilingual posters and flyers were sent to 53 hospitals and ambulance stations in Germany, Norway, and Switzerland for display and distribution. Digital visuals were shared on social media, and medical societies in all three countries were contacted to promote the survey.

#### Study population

Eligible participants were certified EMS professionals with minimum standardized training^1^, including emergency physicians, paramedics, emergency medical technicians, emergency nurses, and trainees, currently or formerly active in prehospital EMS, whether in ground-based ambulances (GEMS), helicopter emergency medical service (HEMS), mountain, or water rescue services. This cohort reflected the target users for the EMS-VAS examined and met the required medical qualifications.

To ensure cross-country comparability, EMS qualifications were grouped into three categories:

- **Category A**: (Emergency) physicians.
- **Category B**: Highest level of vocational or nursing training, or relevant non-medical academic degrees, active in prehospital care.
- **Category C**: Individuals with shorter training programs, trainees, and students.

Credentials were self-reported to preserve anonymity and reduce technical complexity. No verification or tracking (e.g., IP, cookies) was used. Responses were obtained anonymously and participants received no compensation.

Reliable figures on the total EMS workforce were not available for all three countries, which precluded an estimation of the sample’s representativeness relative to the target population. Given the exploratory nature of this study, no a priori sample size calculation was performed. Instead, a convenience sampling approach was adopted, aiming to maximize the number of responses within the available recruitment period.

### Statistical analysis

Data were analyzed using non-parametric methods. Results are reported as median (Mdn), interquartile range (IQR; 25th and 75th percentiles), and Top2 values, with the latter representing the combined proportion of “agree” and “rather agree” responses relative to the total number of responses for each item. For items not answered by all participants, the number of responses included (n) is reported individually. All percentage values were rounded to whole numbers. Unless explicitly stated otherwise, data are reported as pooled, unbalanced values across the entire sample. Comparisons between country groups were performed using the Kruskal-Wallis test (H) with Dunn’s pairwise comparisons applied as post-hoc tests where applicable. The Holm correction was used to adjust for multiple comparisons, as it controls the family-wise error rate. Effect sizes were calculated using epsilon-squared (ε²) and interpreted according to Cohen’s conventions [39]. To assess the association between items measured on ordinal Likert scales, Spearman’s rank-order correlation (r_s_) was applied. Both Kruskal-Wallis test and Spearman’s rank-order correlation were used with the significance level at α = 0.05.

The ‘RStudio Desktop’ development environment (version 2024.09.0 + 375, Posit Software PBC, Boston, MA, USA) and the associated R development language (version 4.4.1, R Foundation for Statistical Computing, Vienna, Austria) were utilized for the analysis. Also, Excel (Version 2408, Microsoft Corp., Redmond, WA, USA) and Datawrapper.de (Datawrapper GmbH, Berlin, Germany) were used.

#### Analysis of free-text responses

Responses to two open-ended question items were analyzed by utilizing ChatGPT’s large language model GPT-5 (OpenAI Inc., San Francisco, CA, USA), accessed on 13 August 2025, as suggested in [40]. As the integration of LLMs into qualitative data analysis is still a relatively young approach, no finalized, universally accepted framework for reporting this analytical method yet exists. Nevertheless, in order to approximate a standardized reporting approach, the reporting in this work is guided by the Consolidated Criteria for Reporting Qualitative Research Guideline to Large Language Models (COREQ + LLM) framework, which is still under development but represents a promising approach to filling this gap [41].

The model used represented the manufacturer’s most current version at the time of the analysis. The choice of provider was due both to existing access to a paid license and to its market leadership at the time. No changes were made to the standard parameter settings. The two prompts used were inductively developed by author CM using a “best effort” approach and were neither peer-reviewed nor formally validated prior to use. They are provided in full in Supplemental Files 2 and 3.

All responses per question item were entered as a single batch along with the instruction prompt in a single analysis run. By doing so, the model was used to facilitate (1) translation of multi-lingual input (preprocessing), (2) inductively identifying recurring themes across all responses (theme generation), (3) assigning each response to the most fitting theme cluster (categorization), and (4) reporting cluster frequencies and sub-facets (summarization). In the absence of inter-rater reliability testing, face validity was ensured through a manual review subsequently conducted by CM. This review included examining the developed theme clusters, spot-checking the cited responses, and verifying the plausibility of the formulated sub-facets. No irregularities requiring further action were identified.

This novel approach of LLM analysis was chosen primarily for efficiency, requiring substantially fewer resources and less time than conventional multi-coder thematic analysis, which would not have been feasible in the scope of this project. Given the small number of free-text responses, this was considered proportionate.

#### Importance analysis of features and non-functional requirements

To identify desired and useful range of EMS-VAS functions, participants rated predefined features with answer options adapted from the Kano model: “I like it”, “I expect it”, “I am neutral” and “I don’t need it” [37]. Features were clustered into four groups of importance adapted from Kano:

- **“Delighter features”**: Strongly supported (≥ 75%), with ≥ 50% liking and with only a low rejection or neutrality rate among users.
- **“Must-Have features”**: Expected essentials whose absence causes dissatisfaction, but their presence does not significantly increase satisfaction. Characterized by majority approval (“Like” or “Expect” > 50%), of which a comparably high proportion expects the feature.
- **“Semi-important features”**: Desired by most, but less critical. Respondents across all countries had a more balanced opinion about these functionalities. They can generate enthusiasm and be well accepted but unlikely to be used or desired by around one-third of the users.
- **“Negligible features”**: Over 50% of respondents rated their availability as unnecessary or responded neutrally.

Throughout the questionnaire, each participant was also asked to prioritize a pre-defined set of so called “Non-Functional Requirements“ (NFR) according to their individual importance. Participants ranked the NFRs from 1 (least important) to 8 (most important), with each rank assigned only once. These individual rankings were summed across participants for each NFR to produce a cumulative score, with higher scores indicating greater overall importance (Eqs. 1 and 2 in Supplemental File 4).

## Results

### Baseline demographics

**Fig. 1:**
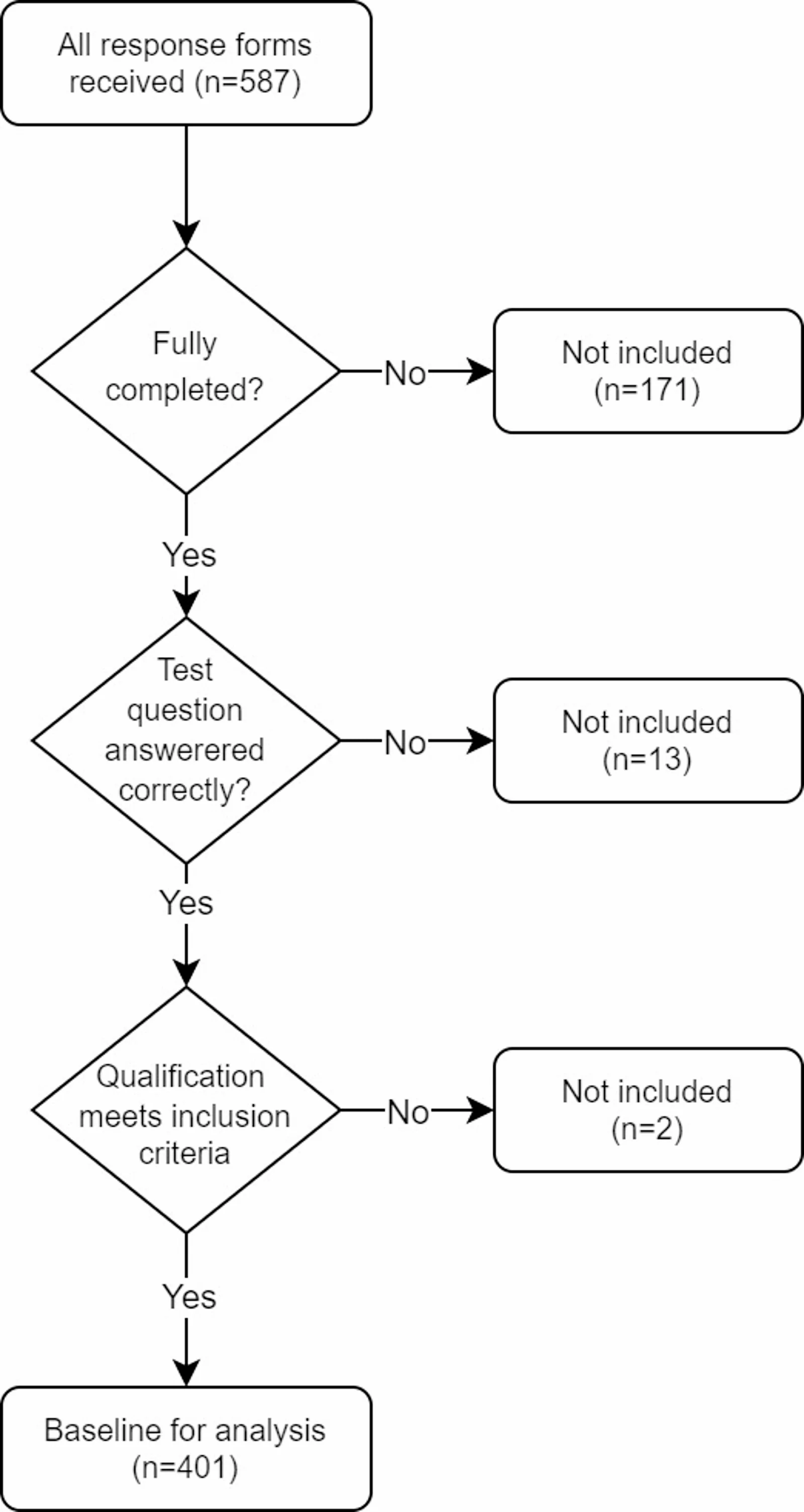
Flowchart of questionnaire response inclusion

In total, 587 response forms were received, of which 186 (32%) were excluded due to incompleteness, incorrectly answered attention check question, and missing professional qualification according to inclusion criteria (Fig. 1). The remaining 401 (68%) responses served as the baseline for further analysis. Of these, 59% were submitted from Germany, 33% from Switzerland, and 8% from Norway.

Table 2.1 and 2.2 present the baseline demographic and professional characteristics of the study sample. Participants had an overall median age of 34 years, with the German group being the youngest and the Norwegian group being the oldest. Three-quarters of the participants were male. According to professional qualification level, respondents were grouped as: category A (10%), category B (70%), and category C (20%).

**Table 2.1 and 2.2:** Overview of demographic characteristics of the study cohort, in total and split per country

	N	%		Median	IQR
**Participants**	401	100%	**Age (in years) (n = 400)**	34	26-44
Germany	235	59%	Germany	31	24-42
Norway	34	8%	Norway	43	37-52
Switzerland	132	33%	Switzerland	37	32-45
**Gender (n = 401)**			**Years of experience (n = 398)**	10	5-20
Male	303	76%	Germany	9	4-19
Female	97	24%	Norway	12	7-20
Diverse	1	0%	Switzerland	12	5-20
**EMS sector (n = 400)**			**Working hours / week (n = 389)**	40	30-45
GEMS	391	98%	Germany	40	22-45
HEMS	21	5%	Norway	36	34-40
Water rescue	10	3%	Switzerland	42	36-45
Mountain rescue	7	2%			
**Professional qualification (n = 400)**					
Category A	39	10%			
Category B	280	70%			
Category C	81	20%			
**Employment status (n = 400)**					
Employed	381	95%			
Volunteering	14	4%			
No longer active in EMS	5	1%			

**Fig. 2:**
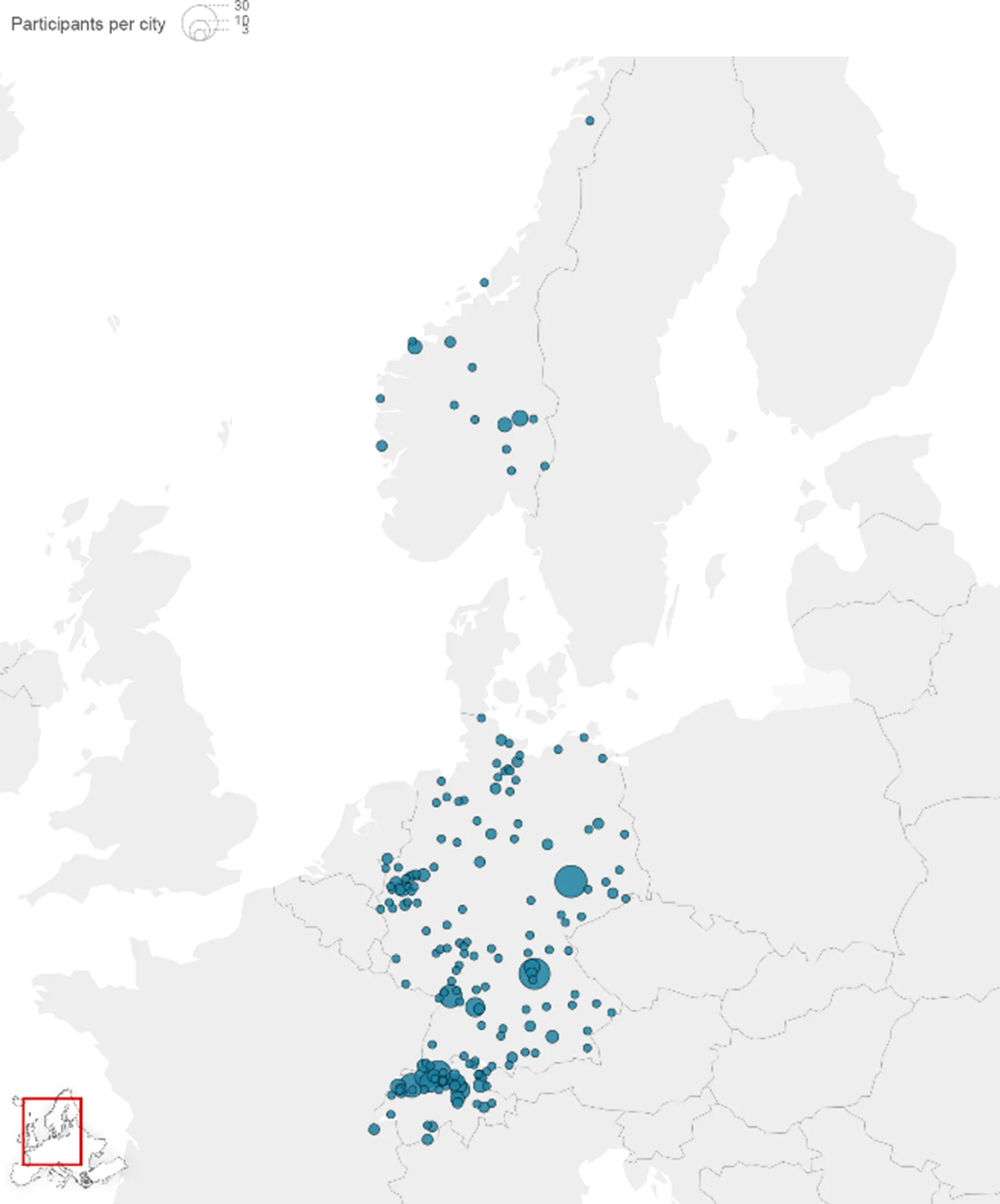
Overview of the participants’ work locations. Circles visualize participants’ places of work. Larger circles indicate more participants from that city

Participants reported on average an EMS experience of 10 years and a 40-hour workweek. The vast majority were employed, while 4% were volunteers and 1% were no longer in EMS. Nearly all work in Ground Emergency Medical Services (GEMS), 5% on helicopters (HEMS), 3% in water rescue, and 2% in mountain rescue (multiple responses were allowed). As Fig. 2 depicts, respondents came from all 16 German states, 19 of the 26 Swiss cantons, and 8 of the 15 Norwegian regions.

### Previous personal experience with AI and voice assistants

Of all participants, 70% assessed themselves as (rather) technologically proficient (median = 2, interquartile range: 2–3, *n* = 400). German respondents scored highest (Top2: 76%) and Norwegian respondents lowest (Top2: 59%). Regarding AI tools such as ChatGPT, 45% reported irregular use, either privately or professionally, 35% had never used them, 17% used them regularly and 3% had discontinued use. A similar pattern was observed for commercial VAS, such as Alexa (Amazon.com, Inc., Seattle, WA, USA) or Siri (Apple Inc., Cupertino, CA, USA), in both professional and private contexts: 38% reported irregular use, 32% had never used them, 20% used them regularly, and 10% had discontinued use.

### Use of digital tools and support systems in EMS

In the EMS context, most of the respondents reported currently using digital solutions for documenting emergency care and mission details on laptop or tablet, with similar prevalence across all countries (Table 3). Systems for pre-notifying hospitals about patients soon to arrive were reported to be used by most German respondents and less common in Norway and Switzerland. Same holds true for applications which monitor and visualize current hospital occupancy. Digital knowledge bases for medical information (e.g., SOPs, drug lists) were more available in Switzerland and Norway than in Germany. Interpreter applications were less common in all countries, with Norway highest, followed by Switzerland and Germany. Telemedicine for remote physician contact and automated tracking of the withdrawal of equipment and consumables were rare, though most frequent in Norway. Overall, four-fifths of the respondents (rather) agreed that previously used digital tools, facilitated their work (Mdn = 2, IQR: 1–2, Top2: 79%, *n* = 394). No substantial differences between countries could be found (H(2) = 1.38, *p* = .502).

**Table 3:** Availability of digital tools in EMS per country

System / Tool	% CH (*n* = 132, 33%)	% DE (*n* = 235, 59%)	% NO (*n* = 34, 8%)	% Total (*n* = 401) (↓)
Digital documentation	77%	89%	79%	84%
Digital medical knowledge base	89%	35%	71%	56%
Hospital patient pre-notification	14%	72%	18%	48%
Hospital occupancy lookup	17%	67%	9%	46%
Interpreter app	23%	17%	38%	20%
Remote physician / Telemedicine	16%	9%	24%	13%
Equipment and consumables tracking	8%	9%	18%	9%
None	2%	3%	6%	3%

Percentage of participants reporting on the use of the listed systems at their EMS station. Sorted descending by overall availability (CH: Switzerland, DE: Germany, NO: Norway)

### Features of an EMS voice assistance system

Participants grouped predefined potential EMS-VAS functionalities into importance clusters, as described in the Methods section. Results are summarized in Table 4.

**Table 4:**
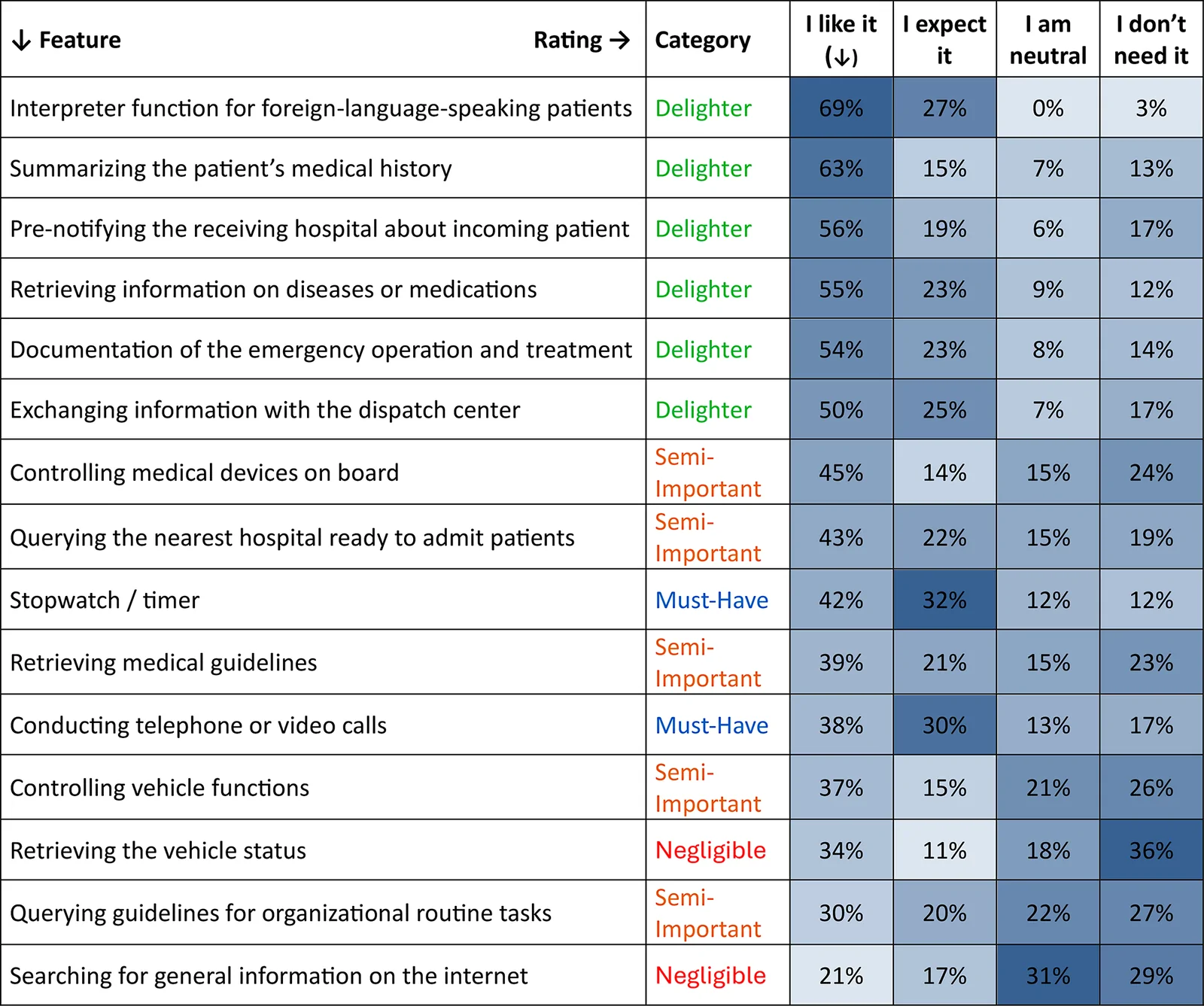
Importance ranking of potential features of an EMS voice assistance system

Participants’ opinion of predefined potential functions of an EMS voice assistance system, highlighted by color according to weighting per column. Sorted descending by share of respondents answering, “I like it”

First, the following functionalities were classified as “Delighter features”:

1. interpreter function for foreign-language-speaking patients in real-time,
2. summarizing the patient’s medical history (while approaching or on scene, using information from national health records or previous reports from EMS or hospital visits),
3. pre-notifying the receiving hospital about the patient soon to arrive (informing the ER of the patient’s personal details and condition, arrival time, and required clinical resources),
4. retrieving information on diseases or medications (e.g., calculation of correct medication doses, toxicological information, and decision support),
5. automated documentation of the emergency operation and treatment, and.
6. exchanging information with the dispatch center (e.g., vehicle and mission status, destination, and requests for additional units).

The interpreter function, in particular, seems to be in high demand, as almost no participants considered it neutral or unnecessary.

Secondly, as desired “Must-Have features” were ranked:

1. Stopwatch / Timer and.
2. conducting telephone or video calls.

The third category comprises “semi-important features”, which include, according to the respondents:

1. Controlling medical devices on board (setting parameters on e.g., patient monitors or ventilators),
2. querying the nearest hospital ready to admit patients (based on the patient’s requirements, travel time and current capacity),
3. retrieving medical guidelines (step-by-step explanation of algorithms, scores and SOPs),
4. controlling vehicle functions (e.g., air conditioning, radio or door lock) and.
5. retrieving guidelines for organizational routine tasks (e.g., disinfection of equipment, material check and donning protective clothing).

As “negligible features” were categorized:

1. Retrieving the vehicle status (e.g., material status and completeness, battery and fuel level or malfunctions) and.
2. Searching for general information on the internet.

Besides the pre-defined set of features clustered before, participants suggested the following additional features to be considered during the development of an EMS-VAS (annotated with the frequency with which the topic was mentioned):

1. Alerting in violence situations (*n* = 3) (triggering an external alarm and sending an emergency message to dispatch via a “code word” (e.g., for aggressive patients)),
2. support during resuscitation (*n* = 2) (e.g., time management, medication reminders, adherence to the correct algorithm during CPR) and.
3. automated informed consent process (*n* = 1) (reading the legally required information aloud to the patient in a standard-compliant manner before the intervention).

### Current availability of EMS voice assistance systems

None of the respondents were aware of any existing EMS-VAS on the market offering a similar range of functions or even a subset of them. However, four participants reported using specialized mobile applications with voice interfaces for isolated tasks during emergency care: “Google Translate” (Google LLC, Mountain View, CA, USA) and “Aidminutes” (aidminutes.org gUG, Hamburg, Germany) for translation (*n* = 1); voice control integrated into GPS systems (*n* = 2); documentation tablets (*n* = 1); and video eyewear (*n* = 1). One respondent each also mentioned using Amazon Alexa and Apple Siri.

### Human-machine-interfaces and non-functional requirements

**Fig. 3:**
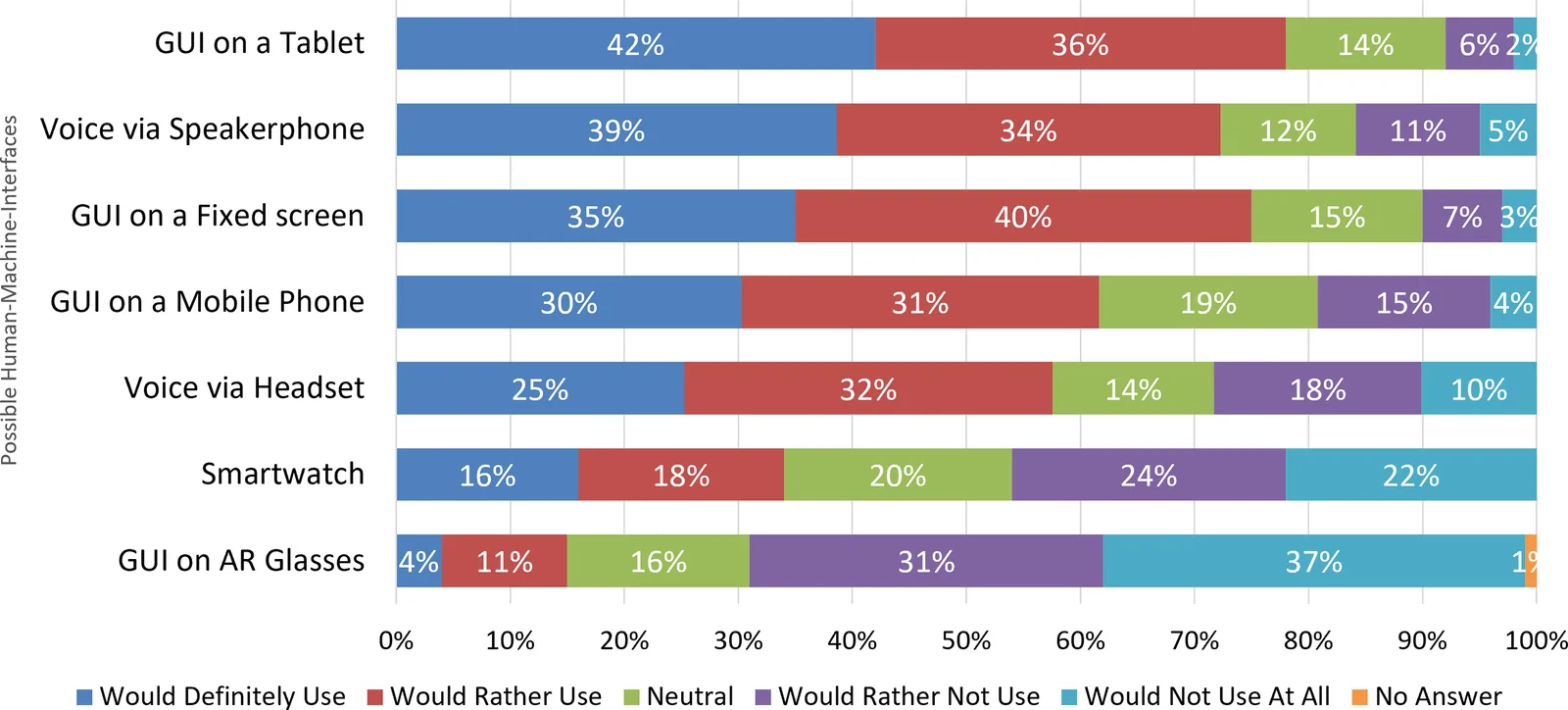
Participants’ opinion on the willingness to use of various human-machine-interfaces to receive system’s output. GUI: Graphical User Interface, AR: Augmented Reality

Figure 3 illustrates the respondents’ opinions on various HMIs through which an EMS-VAS could deliver responses to their previously given voice commands. Participants preferred system output to be displayed on a graphical user interface (GUI) on a large screen, either mobile on a tablet or stationary on a screen mounted in the ambulance.

A GUI on a smartphone would be used slightly less willingly. Receiving the assistant’s responses via synthesized speech would, from a usability perspective, be more likely to occur through a hands-free speaker than through a body-worn headset. Considerably lower average ratings were given to the use of smartwatches and Augmented or Virtual Reality (AR/VR) glasses which were viewed “neutrally” or “rather not” used.

On average, respondents reported a neutral to high level of agreement with the statement that they would be bothered by having to wear a headset or in-ear headphones throughout the emergency mission (Mdn = 3, IQR: 1–4, Top2: 48%).

Participants also rated the priority of predefined non-functional requirements (NFRs). As shown in Table 5, high reliability and usability of an EMS-VAS were rated as most important. Moderate importance was assigned to rapid response to voice commands, interoperability with other operational systems, range of functions provided, and data protection and security. Lower, though still relevant, priority was given to required training and learning effort and to unobtrusive hardware installation within the ambulance vehicle.

**Table 5:** Ranking of non-functional requirements

Non-Functional Requirement (m)	Score (s_m_) (↓)	Share (g_m_)
Reliability	2688	19%
Usability	2618	19%
Response time	2077	15%
Interoperability / Compatibility with other operational systems	1656	12%
Range of functions	1599	11%
Data protection and security	1492	11%
Training and learning effort	1065	8%
Unobtrusive hardware installation	752	5%

Metrics per non-functional requirement calculated as described in Eqs. 1 and 2 in Supplemental File 4, sorted in descending order by score. Higher value indicates higher priority as assessed by the participants

### Practical challenges in deployment

Free-text responses revealed recurring expected challenges in implementing a VAS in EMS, with staff acceptance cited most often (*n* = 58). Resistance was expected among older or less technologically proficient colleagues and was linked to a “we’ve always done it this way” mindset and limited willingness to learn. Concerns included digital overload and skepticism about benefits, suggesting training may face reluctance.

Technical reliability was also strongly highlighted, aligning with NFR findings (*n* = 54). Participants questioned speech recognition in noisy or stressful settings, with dialects, masks, or multiple speakers. System failures and lack of robustness were seen as major risks in critical care.

Costs and financing were noted as relevant challenges, citing high acquisition and maintenance costs, uncertain reimbursement, and questionable cost-benefit as major barriers (*n* = 36). Data protection, surveillance, and legal concerns were also mentioned in 34 responses.

Infrastructure concerns focused on limited network coverage, weak mobile connections, and integration difficulties with existing hospital or ambulance systems (*n* = 20). Patient-related concerns highlighted risks of confusion or reduced trust, especially among older or cognitively impaired patients (*n* = 15).

Further clusters included training and implementation (*n* = 23), emphasizing challenges of adequate education and onboarding overload, and knowledge loss or over-reliance (*n* = 8), where participants feared diminished clinical reasoning due to excessive dependence on technology.

Overall, acceptance, reliability, and cost emerged as the main challenges, with legal, infrastructure, and patient-related issues posing additional critical barriers.

### Privacy and data protection

Privacy and data protection were considered (rather) important by 59% of respondents when it comes to implementing an EMS-VAS (Mdn = 2, IQR: 1–3, Top2: 59%, *n* = 399). A statistically significant difference in this assessment between the three countries could be observed (H(2) = 24.07, *p* <.001), with a medium effect size (ε² = 0.06).

In the free-text responses on data privacy, unauthorized access to sensitive health data was mentioned as the most common concern, encompassing risks such as hacking, data loss, misuse, interception, manipulation, and commercial exploitation, often attributed to weak encryption (*n* = 84). Cloud-based systems, especially those run by large tech companies, were deemed particularly vulnerable. A closely related concern was the risk of sensitive patient information being overheard by unauthorized individuals (*n* = 79). Situations included treatment in public spaces, inside ambulances, or near bystanders, family, or other personnel. Loudspeaker use, speaking loudly in noisy environments, and continuous listening modes were seen as exacerbating factors.

Concerns about data storage location and jurisdiction reflected mistrust of foreign servers and jurisdictions with weaker privacy standards, particularly third-party cloud providers (*n* = 32). Legal and regulatory requirements, such as GDPR, were seen as both essential safeguards and significant barriers, with strict or inflexible interpretations perceived as delaying or preventing deployment (*n* = 33). Technical reliability and security formed a distinct theme, including doubts about encryption strength, interface security, and system accuracy under operational stress (*n* = 28).

Ethical and legal concerns focused on rights, consent, and accountability - particularly challenges in obtaining informed consent during emergencies, assigning liability for breaches, and ensuring secure user authentication (*n* = 17).

Fifteen responses raised concerns about continuous monitoring and tracking, fearing that persistent recording could enable detailed surveillance of patients and staff, potentially leading to misuse in performance evaluations or legal proceedings.

### Assessment of impact

Overall, three-quarters of the participants agreed with the concept of an EMS-specific VAS, stating they would likely use it during emergency response calls if available in their ambulance (Mdn = 2, IQR: 1–3, Top2: 74%). A weak, statistically significant positive correlation was found between anticipated likelihood of use and self-assessed technology affinity (r_s_(398) = 0.25, *p* <.001, 95% CI [0.16, 0.34]). No significant correlation was found between anticipated likelihood of use and the reported age (r_s_(398) = -0.07, *p* = .166, 95% CI [-0.17, 0.03]).

**Fig. 4:**
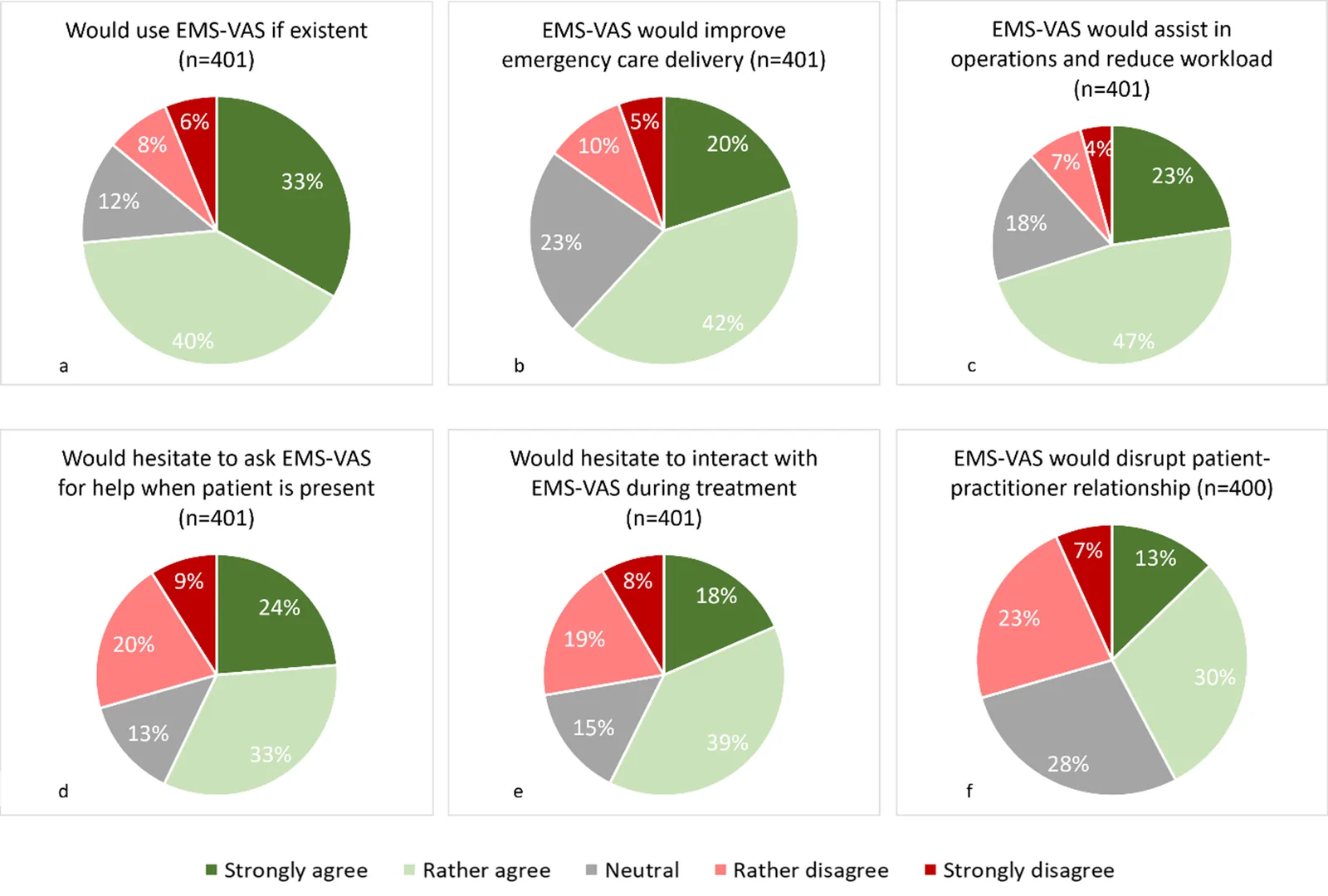
**a-f**: Participants’ views on impact and implications for real-world use

Most participants reported being (rather) hesitant to interact with an EMS-VAS during emergency treatment (Mdn = 2, IQR: 2–4, Top2: 57%) or to ask for help while patients are present (Mdn = 2, IQR: 2–4, Top2: 57%) (Fig. 4). They also expressed neutrality on preferring speech-based documentation over paper or tablet (Mdn = 3, IQR: 2–4, Top2: 50%, *n* = 400).

Most respondents (rather) believed a functioning EMS-VAS could improve emergency care (Mdn = 2, IQR: 2–3, Top2: 62%). Interest in contributing to development (e.g., consulting or testing) was more neutral (Mdn = 3, IQR: 2–4, Top2: 47%, *n* = 398), with no notable country differences (H(2) = 5.36, *p* =.068).

Uncertainty remains about whether an EMS-VAS would disrupt patient-provider relationships (Mdn = 3, IQR: 2–4, Top2: 42%, *n* = 400). Most respondents tended to agree that an EMS-VAS would likely reduce their workload during shifts (Mdn = 2, IQR: 2–3, Top2: 70%).

## Discussion

The results demonstrated the potential of specialized voice assistance systems in EMS. Emergency personnel are already supported by various technical tools today, whose availability varies by country, and most respondents reported that these tools reduce their workload. Three-quarters of respondents could envision using an EMS-VAS, though not all proposed functionalities were considered equally important and practical concerns were mentioned. Traditional graphical interfaces remained important for receiving system-generated information. Translating this concept into practice will also require fostering user acceptance, clarifying cost coverage, and addressing data protection concerns.

### Study context and comparability with existing research

The dataset spanned a broad geographical area and reflected a demographic profile similar to previous studies of EMS personnel (predominantly young, male, and highly vocationally trained) [2, 42]. Respondents reported being unaware of any EMS-VAS currently in use that resembles the presented concept and covers a similar range of functionalities. This is consistent with the preliminary literature review and the scoping review by Hedderson et al. [28] and suggests a gap between user demand and the availability of a ready-to-use system for voice assistance. In the absence of similar large-scale surveys on voice-enabled assistance systems in prehospital care, this study established a basis for future research, while being unable to directly compare to user requirements in similar work. However, participants emphasized reliable and user-friendly operation as fundamental requirements for medical devices, especially in stressful environments, which is in line with previous studies in neighboring fields [23, 28, 43].

### Implications for clinical translation

Although the study followed a hypothetical design, focusing on the evaluation of a purely conceptual construct, implications for future translation into clinical practice can be drawn and discussed based on the results.

First, several desired EMS-VAS functionalities require integration with external systems. For instance, generating a summary of a patient’s medical history depends on access to nation-wide electronic health records (EHR), which are at different stages of implementation across the surveyed countries [44]. Nations already having well-proven, wide-spanning EHRs such as Estonia or Denmark could particularly benefit from EMS-VAS: all patient data are centrally available and can be quickly filtered, adapted to the current case and delivered in a user-friendly manner via voice during emergencies [45, 46]. Other functionalities aim to address existing clinical challenges. Automatically creating treatment documentation from voice interactions could reduce information loss, especially during intersectoral handovers. Evans et al. reported that only 75% of information transferred from EMS to ER staff was documented in writing, with omissions primarily concerning prehospital treatment [47].

Second, as user acceptance was seen as a key prerequisite when it comes to operational deployment, its continuous evaluation and maximization can be considered crucial for success. To achieve this, clearly demonstrating added value and enhancing the perceived ease of use will help to motivate users to invest the effort required to learn its proper use, as also suggested by AlQudah et al. [48]. This is especially important concerning users with lower technology affinity, who should be given special consideration. Doing so also includes addressing privacy concerns, both on the patient side (fear of unauthorized access to sensitive health data) and on the staff side (fear of surveillance). Particular attention should be paid to ensure that an EMS-VAS reduces, rather than increases, the cognitive overload during emergency operation that respondents partially reported experiencing today. Development should therefore be conceived as digitalization (comprehensive process transformation through native digital data) rather than mere digitization (converting existing analog processes into digital form).

Third, respondents also highlighted reliable and user-friendly operation. When interacting with a voice assistant, participants reported preferring to receive information via graphical interfaces (e.g., screens or tablets) rather than speech alone. Newer interfaces like AR glasses were viewed less favorably, possibly due to limited familiarity and anticipatory rejection [49]. This assessment may change once modern HMIs become more familiar to users through wider adoption in EMS practice.

Fourth, while beyond the scope of this work, regulatory frameworks resulting from the intended use and functional scope of an EMS-VAS must not be neglected. These include medical device regulations, requirements arising from AI legislation (e.g., the EU AI Act), and provisions for critical infrastructure. As this technology is novel and may set a precedent, such considerations should be addressed early in the development process. Given its early-stage status, an EMS-VAS would first need to undergo the relevant regulatory certification processes before being suitable for practical use.

### Limitations

This study is subject to several limitations. First, country contribution was uneven: Germany accounted for nearly 60% of the sample, Norway only about 10%. Where contextually relevant, results were analyzed separately by country group; pooled analyses, however, still overrepresent German participants’ opinions, leading to a geographic sampling bias.

Second, convenience sampling without demographic quotas introduced selection bias, limiting generalizability to the broader EMS population; results were therefore only reported descriptively. Respondents likely showed above-average technological proficiency, as tech-affine individuals are more likely to participate in online surveys, potentially inflating acceptance of the concept. Physician participation was also lower than expected - notable given Germany’s physician-centered prehospital model - introducing a non-response bias. This may reflect weaker distribution support, limited perceived benefits, or the often part-time nature of physicians’ EMS roles.

Third, the LLM-based approach used to support qualitative analysis of the open-ended questions exhibits certain limitations. While LLMs offer considerable advantages for scientific purposes, their probabilistic architecture inherently carries certain risks, including the amplification of biases present in the training data, hallucinations, indeterministic output and a limited understanding of nuanced contexts [41, 50–52]. Two specific limitations apply to the present analysis: All responses per question were processed in a single execution run as mentioned in the Methods section. Hence, output stability across repeated runs was not formally evaluated, leaving the reproducibility of the resulting thematic structure uncertain. In addition, while manual human review ensured face validity, it does not substitute for coding reliability, as no independent human coding, inter-rater agreement assessment, or systematic comparison between human- and LLM-generated themes was performed. The analysis therefore relies on a single coding approach without formal reliability assessment.

Fourth, this study used an “everything is possible” approach, presenting the EMS-VAS concept hypothetically. As the concept was entirely novel to the participants, findings could not draw on prior experience, prototype testing, or practical implementation aspects, and perceptions of certain items may have varied based on individual interpretation.

## Future directions

Practical recommendations can be derived from the results, which should be considered in the future development and deployment of an EMS-VAS:

1. Ensure a high level of usability and reliability (in terms of Word Error Rate, connectivity and power failure safety, etc.).

2. Prioritize core (“Must-Have”) and high-value (“Delighter”) features.

3. Focus on fewer, well-functioning features rather than many poorly implemented ones.

4. Identify and integrate external services and interfaces where useful and motivate for interoperability where necessary.

5. Use an iterative development approach and regularly incorporate feedback from users and other stakeholders.

6. Design for intuitive user experience that requires minimal training.

7. Educate users about advantages for their daily work, functional limitations, and privacy aspects.

Future research should focus on hands-on user testing of EMS-VAS prototypes developed from the findings of this study. From a healthcare research perspective, it should also be investigated to what extent supporting users in individual tasks impacts quality of care or employee satisfaction. Lastly, user opinions from other countries and continents are desirable, given that EMS exist worldwide and all populations should have access to this kind of innovation.

## Conclusion

Prehospital emergency care faces increasing challenges from demographic shifts, rising patient volumes, and increasingly complex treatments. While several digital, screen-based applications already exist to support staff during emergency missions, voice assistance systems for emergency medical services (EMS-VAS), as introduced in this paper as a hypothetical concept, could represent the next step in the evolution of such aids. The results from Germany, Norway, and Switzerland indicate that such a system may support users in communication, documentation, and information retrieval. Highly desired features included language translation, patient history summaries, hospital pre-notification, treatment documentation, and dispatch integration. Respondents emphasized reliability, ease of use, and data security as critical requirements to achieve high user acceptance. There is substantial willingness to adopt voice assistants in EMS if they meet user needs and regulatory standards. Future research should focus on prototype development, real-world testing, and international user feedback to ensure practical implementation and acceptance.

## Supplementary Information

Below is the link to the electronic supplementary material.

Supplementary Material 1

Supplementary Material 2

Supplementary Material 3

Supplementary Material 4

## Data Availability

The datasets generated and analyzed during the current study are available in the Zenodo repository, https://doi.org/10.5281/zenodo.18327568.

## Footnotes

1 following the respective national requirements, in Germany at least “Rettungssanitäter“, in Norway “Ambulansefagarbeider“ or “Sykepleier“, in Switzerland “Transport assistant / Transporthelfer“.

## Abbreviations

AI: Artificial Intelligence
AR/VR: Augmented/Virtual Reality
ASR: Automatic Speech Recognition
CH: Switzerland
CPR: Cardiopulmonary Resuscitation
DE: Germany
EHR: Electronic Health Record
EMS: Emergency Medical Services
ER: Emergency Room
FTE: Full-Time Equivalent
GEMS: Ground-based Emergency Medical Services
GUI: Graphical User Interface
HEMS: Helicopter Emergency Medical Services
HMI: Human-Machine-Interface
IQR: Interquartile Range (25%-75%)
LLM: Large Language Model
Mdn: Median
NFR: Non-Functional Requirement
NO: Norway
OECD: Organization for Economic Co-operation and Development
SOP: Standard Operating Procedures
VAS: Voice Assistance Systems
