# Supplementary material for: A multinational cross-sectional survey on the use of AI-based voice assistance systems in emergency medical services
Source: Scand J Trauma Resusc Emerg Med. 2026 Sep 15;34:155. doi: 10.1186/s13049-026-01695-1 (PMC13599295; doi:10.1186/s13049-026-01695-1)
Supplement: Supplementary file 1 [file 13049_2026_1695_MOESM1_ESM.pdf]

Möllenhoff, C. et al.: An International Cross-Sectional Survey on Use of AI-based Voice Assistance Systems in Emergency Medical Services

Supplement 1

Figure 4: Printout of the international online questionnaire

Legend Figure 4: Printout of the online questionnaire used in the survey in the English version for Switzerland, which (with only a few adjustments to individual questions to reflect country-specific characteristics) was translated into three languages for all participating countries.

[Pictures start on following page]

Voice Assistance Systems in Emergency Medical Service (CH)

Welcome and thank you for your interest in this international survey!

Participation will take approximately 10 minutes.

Every participation is important!

**Important notes at the beginning**<br>(please read before starting)	1.) The survey is conducted **anonymously**. Providing a name is not required, and we do not store any other information from you. Participation is also **voluntary**.<br>2.) Participation is open to **all employees (paid or voluntary)** in the Swiss Emergency Medical Service who have **at least the qualification of Ambulance Technician with federal certificate / Technicien Ambulancier (TA) / Transportsanitäter/in mit eidg. Fachausweis** (TranspSan/TA, RettSan Hf / AD, Anaesthesia nurse, Emergency Physicians). Excluded are first responders, transport assistants, etc., as well as doctors and nurses without active roles in the emergency services.<br>3.) This survey is being conducted in three countries (Germany, Norway, Switzerland). This is the **Swiss questionnaire**. If you work in one of the other countries, please select the correct questionnaire at rescue-research.de (https://rescue-research.de). If you want to fill in the Swiss study but would like to use the questionaire in German language, you have the possibility to **switch languages** in the respective filed in the upper part of this page.<br>4.) **Every answer is important!** However, for the subsequent evaluation, only questionnaires that have been completed to the end can be considered. Therefore, please complete the survey **in full until the end**! You have the option to deny your anwers to certain questions. If you wish to stop during the process, click on the text in the top right corner.<br>5.) There are **no right or wrong answers**. What is asked for is always your own opinion.<br>6.) Each participant may **only participate once** in the survey.<br>7.) Participation is possible **until Sunday, 8th September 2024**.<br>8.) At the end of the questionnaire, you can voluntarily and without associating your responses, sign up for the **results mailing list**.<br>9.) Please read the **data protection notice** below before starting.

**About the study**	The online questionnaire you are about to start is aimed at researching how new technologies can be beneficially used in the emergency services. This project specifically involves the **development of a voice assistance system for ambulances**, which, similar to Alexa, Siri, or Cortana at home, assists emergency personnel during operations. More information will be provided during the survey.<br>Your participation is crucial as users (you!) are involved from the beginning in the development process!<br>The results of the questionnaire will be published in a scientific work and made accessible to you.<br>For more detailed information about the project, visit www.rescue-research.de/about-the-project/ (https://rescue-research.de/about-the-project/)
**Data protection notice**	Please read our data protection notice at www.rescue-research.de/privacy/ (https://rescue-research.de/privacy/) before starting.<br>By clicking the "Next" button, you agree to these terms.

This survey is conducted by the Innovation Center Computer Assisted Surgery at the Medical Faculty of the University of Leipzig.
Contact: moellenhoff@rescue-research.de

There are 35 questions in this survey.

Demographic Data

How old are you? *

❗ Only an integer value may be entered in this field.
Please write your answer here:

Please enter your age **in years**.
If you **do not wish to answer**, please enter 100.

What is your gender? *

❗ Choose one of the following answers
Please choose **only one** of the following:

- ◯ female
- ◯ male
- ◯ divers
- ◯ No answer

What is your native language? *

❶ Choose one of the following answers
Please choose **only one** of the following:

- ◯ German
- ◯ Swiss German
- ◯ French
- ◯ Italian
- ◯ Rhaeto-Romanic
- ◯ English
- ◯ Spanish
- ◯ Norwegian
- ◯ Danish
- ◯ Swedish
- ◯ No answer
- ◯ Other ______

Details of work in the Emergency Medical Service

What is your **highest qualification in emergency medical service**? *

❶ Choose one of the following answers
Please choose **only one** of the following:

◯ Paramedic with diploma / Ambulancier diplômé (AD) / Rettungssanitäter/in Hf

◯ Ambulance Technician with federal certificate / Technicien Ambulancier (TA) / Transportsanitäter/in mit eidg. Fachausweis

◯ Transport assistant / Transporthelfer

◯ Anaesthesia nurse / Anästhesiepfleger/in

◯ Emergency physician / Notarzt SGNOR

◯ No answer

◯ Other [ ]

Are you currently working in the emergency medical service on a **voluntary** or **employment** basis?

*

❶ Choose one of the following answers
Please choose **only one** of the following:

◯ Employed

◯ Voluntary

◯ Currently no longer working in EMS

◯ No answer

"Employment" includes any type of professional/paid work in the emergency medical service, whether full-time or part-time. If you are **both a volunteer and a employed worker** , please select "employed".

How many **years** have you worked (in total) in the emergency medical service so far? *

❗ Only an integer value may be entered in this field.
Please write your answer here:

Please enter the **exact number** or **round** to the nearest 5 if necessary.
If you **do not wish to answer**, please enter 100.

How many **hours** do you currently work **weekly** in the emergency medical service? *

❗ Only numbers may be entered in this field.
Please write your answer here:

Please enter the **exact number** or **round** to the nearest 5 if necessary.
If you **do not wish to answer**, please enter 100.

In which **divisions** do you currently work in the ambulance service? *

Check all that apply

Please choose **all** that apply:

- [ ] Ground-based Ambulance Service (with cars)
- [ ] Air Ambulance (with planes and helicopters)
- [ ] Water Rescue (with boats)
- [ ] Mountain Rescue
- [ ] No answer
- [ ] Other: ______

In which **city** do you work in emergency medical service? *

Please write your answer here:

______

If you work in **multiple cities**, please indicate the city where you primarily work. If you have multiple jobs and therefore work **part-time** or **voluntarily** in the emergency medical service, please indicate the city where you work in the EMS. If you **do not wish to answer**, please enter 100.

In which **canton** is the city where you work located? *

❶ Choose one of the following answers
Please choose **only one** of the following:

- ◯ Aargau / Argovie
- ◯ Appenzell A.Rh. / Appenzell Rhodes-Extérieures
- ◯ Appenzell I.Rh. / Appenzell Rhodes-Intérieures
- ◯ Basel-Landschaft / Bâle-Campagne
- ◯ Basel-Stadt / Bâle-Ville
- ◯ Bern / Canton de Berne
- ◯ Freiburg / État de Fribourg
- ◯ Genf / Genève
- ◯ Glarus / Glaris
- ◯ Graubünden / Grisons
- ◯ Jura
- ◯ Luzern / Lucerne
- ◯ Neuenburg / Neuchâtel
- ◯ Nidwalden / Nidwald
- ◯ Obwalden / Obwald
- ◯ Schaffhausen / Schaffhouse
- ◯ Schwyz
- ◯ Sankt Gallen / Saint-Gall
- ◯ Solothurn / Soleure
- ◯ Tessin
- ◯ Thurgau / Thurgovie
- ◯ Uri
- ◯ Wallis / État du Valais
- ◯ Waadt / Canton de Vaud
- ◯ Zug / Zoug
- ◯ Zürich / Zurich
- ◯ No answer

Previous Experience in Technology and Assistance Systems

How **technology-interested** would you describe yourself in general? *

❶ Choose one of the following answers
Please choose **only one** of the following:

- ◯ Very technology-interested
- ◯ Rather technology-interested
- ◯ Neutral
- ◯ Rather not technology-interested
- ◯ Not technology-interested at all
- ◯ No answer

Please rate how interested and competent you generally consider yourself in using technology (such as computers, smartphones, wearables, smart speakers, etc.)!

Do you use a digital **voice assistant** privately or professionally?

(for example, on your phone or at home as a device, such as Alexa, Siri, Cortana, Google Assistant, or Samsung Bixby) *

❶ Choose one of the following answers
Please choose **only one** of the following:

- ◯ Regularly
- ◯ Unregularly
- ◯ Don't use it anymore
- ◯ Never used
- ◯ I don't know
- ◯ No answer

Do you use **AI applications** privately or professionally? *(e.g., OpenAI ChatGPT or DALL-E, Beautiful.ai, etc.)* *

❶ Choose one of the following answers
Please choose **only one** of the following:

- ◯ Regularly
- ◯ Unregularly
- ◯ Don't use it anymore
- ◯ Never used
- ◯ I don't know
- ◯ No answer

General Questions on Emergency Medical Service

How serious do you currently consider the following mentioned **problems for emergency medical services in Switzerland** to be? We are asking for your personal opinion. *

Please choose the appropriate response for each item:

	Very problematic	Rather problematic	Neutral	Rather not problematic	Not problematic at all	No answer
**Staff shortage**	○	○	○	○	○	○
**Too much bureaucracy / administrative burden**	○	○	○	○	○	○
**Please click "No answer" here! (for verification purposes)**	○	○	○	○	○	○
**High workload due to many emergency responses per shift**	○	○	○	○	○	○
**Lack of health literacy among the population**	○	○	○	○	○	○
**Insufficient equipment in emergency vehicles**	○	○	○	○	○	○
**Missing or inadequate digitalization**	○	○	○	○	○	○
**Insufficient training of staff**	○	○	○	○	○	○
**Restricted medical authority for paramedics/EMTs**	○	○	○	○	○	○
**Overcrowded emergency rooms and hospitals**	○	○	○	○	○	○

	Very problematic	Rather problematic	Neutral	Rather not problematic	Not problematic at all	No answer
**Weak salaries**	○	○	○	○	○	○

Which of the following **systems** are available to you at your emergency station? *

❶ Check all that apply

Please choose **all** that apply:

☐ Electronic Patient Journal / Documentation on tablet or laptop

☐ Digital pre-notification of hospital staff with arriving patient details

☐ Overview of available hospital ressources ("Which hospitals are ready to receive patients?")

☐ Telemedical consoltation of remote physicians/experts (with audio/video chat)

☐ Digital medical reference books for lookup (e.g. sample algorithms, guidelines, etc.)

☐ Digital material management and consumption recording in the vehicle (Automated creation of a list of used material and devices)

☐ Translator service for foreign-language patients

☐ None of the systems mentioned are available

☐ No answer

☐ Other: ____________

If you **work at multiple stations**, indicate that a system is available if it is present at at least one station.

Are there any additional **tasks or processes** that you typically handle during an emergency response where you would wish for digital support?

Please write your answer here:

Are there any **information or data** that you would like to have available in real-time during every emergency response, but currently do not receive or only obtain with significant manual effort? (If yes, from where?)

Please write your answer here:

Please rate your agreement with the following statement: "I find that the **digital tools** I have used **so far** in emergency medical services **assist me in my work** and **reduce my workload**." *

❶ Choose one of the following answers

Please choose **only one** of the following:

- ◯ Strongly agree
- ◯ Rather agree
- ◯ Neutral
- ◯ Rather disagree
- ◯ Strongly disagree
- ◯ No answer

Information text

Please briefly read the following text before proceeding!

This survey is about so-called **voice assistance systems** (hereinafter referred to as "voice assistants").

Voice assistants are devices equipped with a microphone, speaker, possibly a screen, and associated software that can recognize human speech and perform tasks based on that input. Users **utilize their voice** for questions or commands instead of typing them as usual, and receive **responses through artificially generated speech or displays** on graphical interfaces. Voice assistants can be used, for example, to answer questions, control devices, or provide information. You may be familiar with voice assistants for home use, such as Amazon Alexa, Apple Siri, Microsoft Cortana, or Google Assistant.

When answering the following questions, please think of such a system specifically designed for use in emergency medical service and supporting related tasks!

The system might be called "Medix," and you could ask questions or give commands to the assistant by saying, "Medix, answer my question xyz!"

Such a solution does not yet exist in practice, but your responses will help advance the development of such an emergency medical service voice assistant.

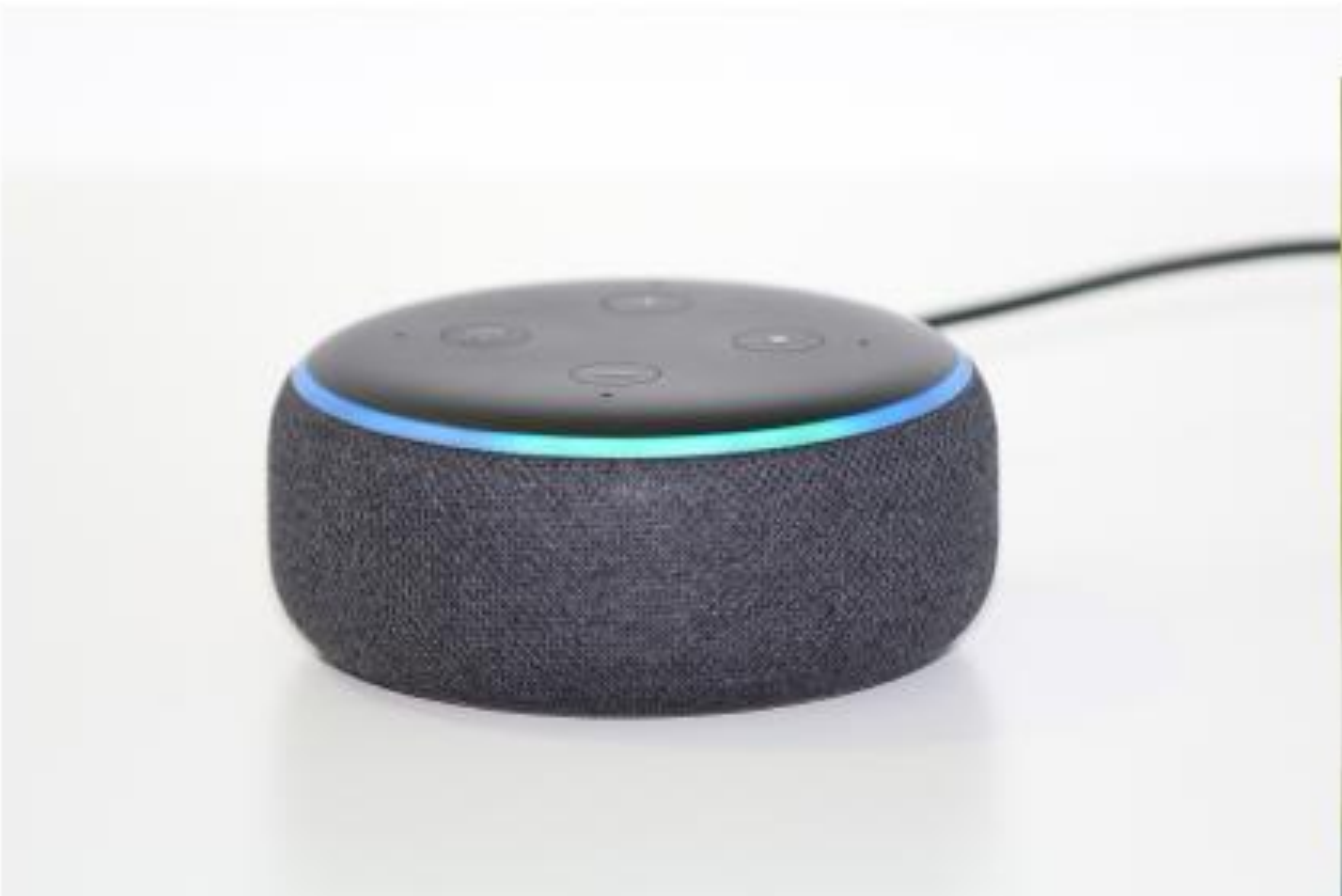
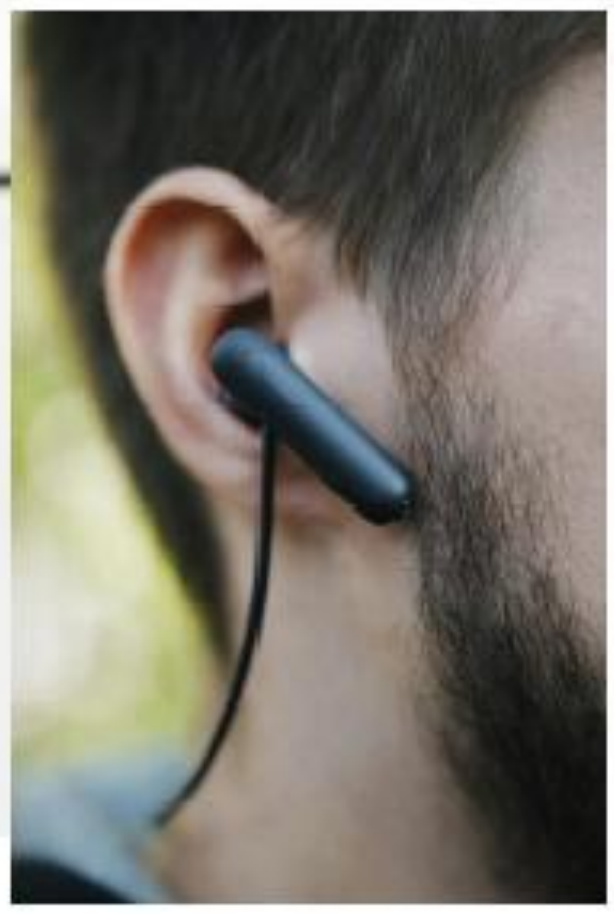

Questions about Voice Assistants: Functions

This section may seem overwhelming due to the amount of text, but **it can be completed quickly!**

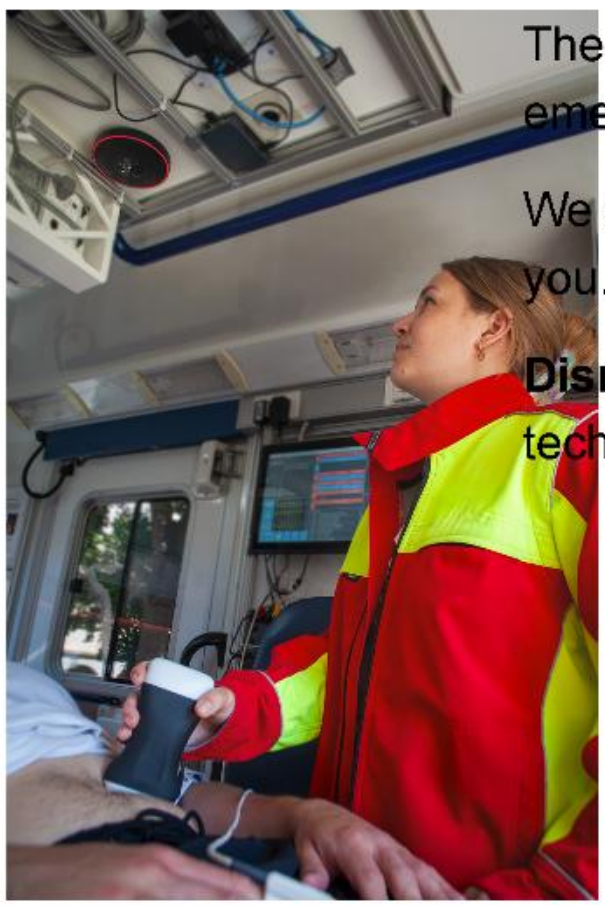

The following table lists **possible features** of a voice assistant for emergency medical services.

We are interested in knowing **how important** these features are to you. For each feature, select the response that best applies.

Disregard whether these features can be practically, legally, or technically implemented. Imagine everything is possible.

What would you say if an **emergency service voice assistant** had the following **features**? *

Please choose the appropriate response for each item:

	1- I like that	2 - I expect that	3 - I am neutral	4 - I don't need that / I would not use that	No answer
**[1] Query of the next available hospital (e.g. "Medix, what is the nearest available hospital suited for my patient?")**	○	○	○	○	○
**[2] Pre-notify the destination hospital staff about the incoming patient (e.g. "Medix, pre-register us with the Emergency Room at the University Hospital Leipzig!")**	○	○	○	○	○
**[3] Exchange of information with the dispatch center (Requesting additional vehicles, specifying destination, etc.) (e.g. "Medix, request an additional emergency doctor for analgesia!")**	○	○	○	○	○

	1- I like that	2 - I expect that	3 - I am neutral	4 - I don't need that / I would not use that	No answer
**[4] Querying medical guidelines (as a step-by-step dialogue) (e.g. "Medix, what is the algorithm for anaphylaxis?")**	◯	◯	◯	◯	◯
**[5] Querying information about diseases or medications (e.g. "Medix, is a soy allergy a contraindication for administering propofol?")**	◯	◯	◯	◯	◯
**[6] Querying guidelines for routine tasks (Disinfection, material check, infection protection, etc.) (e.g. "Medix, guide me through the material check!")**	◯	◯	◯	◯	◯
**[7] Documentation of the emergency operation (e.g. "Medix, document the application of a pelvic sling now!")**	◯	◯	◯	◯	◯

	1- I like that	2 - I expect that	3 - I am neutral	4 - I don't need that / I would not use that	No answer
[8] Control of medical devices such as patient monitors or ventilators (e.g. "Medix, set the blood pressure measurement interval to 5 minutes!")	○	○	○	○	○
[9] Control of vehicle functions (temperature/ AC, lighting, locking,...) (e.g. "Medix, turn on all the lights in the patient room!")	○	○	○	○	○
[10] Translation function for foreign language patients (e.g. "Medix, ask the patient in Spanish about their symptoms!")	○	○	○	○	○
[11] Querying the vehicle status (Is the material complete? Is the vehicle ready to go?,...) (e.g. "Medix, is the material complete?")	○	○	○	○	○
[12] Making phone or video calls (e.g. "Medix, make a video call to the tele-emergency doctor!")	○	○	○	○	○

	1- I like that	2 - I expect that	3 - I am neutral	4 - I don't need that / I would not use that	No answer
**[13] Summarizing the patient's history from the medical record (e.g. "Medix, did the patient have any hospital stays in the last year?")**	○	○	○	○	○
**[14] Stopwatch / Timer (e.g. "Medix, set the timer for 30 seconds!")**	○	○	○	○	○
**[15] Searching for general information on the internet (e.g. "Medix, will it rain this afternoon?")**	○	○	○	○	○

Can you think of any **additional features** you would want from such a voice assistant?

Please write your answer here:

With an assistance system, information can be input or output through **various devices / human-machine interfaces**.
For example, you might ask the system a question via voice command and then receive an answer.

Which **interfaces** would you like to use for this interaction?
*

Please choose the appropriate response for each item:

	1 - I would definitely use it	2 - I would rather use	3 - Neutral	4 - I would rather not use it	5 - I would not use it at all	No answer
**Voice via hands-free device / loudspeaker**	○	○	○	○	○	○
**Voice via headset**	○	○	○	○	○	○
**Graphical interface on display in the vehicle**	○	○	○	○	○	○
**Graphical interface on tablet**	○	○	○	○	○	○
**Graphical interface on tablet**	○	○	○	○	○	○
**Smartwatch**	○	○	○	○	○	○
**AR/VR glasses**	○	○	○	○	○	○

AR glasses ("Augmented Reality") are data glasses that can be described as "oversized sunglasses with a screen." The user sees the entire environment through the glasses but also has graphical content projected into their field of view through special technology (see image). **VR glasses** ("Virtual Reality") work similarly, but the environment is displayed on screens in front of the eyes instead of through a semi-transparent glass.

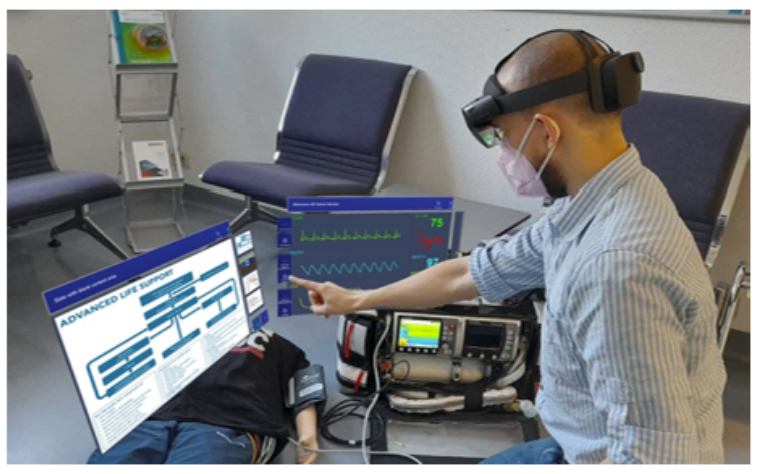

Do you know of an **existing voice assistant** for emergency medical services that possesses any or similar features to those listed above? *

❶ Choose one of the following answers

Please choose **only one** of the following:

- ◯ Yes
- ◯ No
- ◯ No answer

Please provide the **name**, **manufacturer**, or **link** to the voice assistant for emergency medical services that you are aware of below!

Only answer this question if the following conditions are met:
Answer was 'Yes' at question '23 [bekanntesa]' (Do you know of an existing voice assistant for emergency medical services that possesses any or similar features to those listed above?)

Please write your answer here:

Questions about Voice Assistants: General

Please **rate** the following **statements**! *

Please choose the appropriate response for each item:

	Strongly agree	Rather agree	Neutral	Rather disagree	Strongly disagree	No answer
**I would find it strange / I would hesitate to interact with the voice assistant during patient treatment.**	○	○	○	○	○	○
**I would find it strange / I would hesitate to ask the voice assistant for help or information during patient treatment.**	○	○	○	○	○	○
**It would bother me to wear a headset or in-ear headphones during an entire emergency operation.**	○	○	○	○	○	○
**I would prefer a voice-based documentation solution over writing on paper or tablet.**	○	○	○	○	○	○

Please rank the following **requirements** for a voice assistance system in terms of their importance to you!

❶ All your answers must be different and you must rank in order.

❶ Please select at most 8 answers

Please number each box in order of preference from 1 to 8

[ ] Easy usability

[ ] Reliability

[ ] Range of functions

[ ] Data protection and security

[ ] Fast response of the system to voice commands

[ ] Training / learning effort

[ ] Compatibility with other operational systems

[ ] Discreet installation of the hardware

How important is **data protection** to you when using an emergency service voice assistant in a professional setting? *

Choose one of the following answers
Please choose **only one** of the following:

- ◯ Very important
- ◯ Rather important
- ◯ Neutral
- ◯ Rather unimportant
- ◯ Not important at all
- ◯ No answer

What are your **specific concerns or worries** regarding **data protection** in connection with an emergency service voice assistant?

Only answer this question if the following conditions are met:
Answer was 'Very important' *or* 'Rather important' *or* 'Neutral' *or* 'Rather unimportant' at question '27 [datenschutz]' (How important is data protection to you when using an emergency service voice assistant in a professional setting?)

Please write your answer here:

How important is it to you to have the opportunity to **actively participate** in the development of such a system?
(e.g., providing professional advice on features, testing prototypes, etc.)? *

Choose one of the following answers
Please choose **only one** of the following:

- Very important
- Rather important
- Neutral
- Rather unimportant
- Not important at all
- No answer

Do you foresee any **problems or challenges** with the introduction of an emergency service voice assistant on the ambulance vehicles at your station? If yes, what are they?

Please write your answer here:

Frage zu Sprachassistenten: Final questions

Almost done!
Just a few more questions to finish up!

How **likely** are you to **use** a voice assistance system (as described in this survey) during your emergency operations? *

Choose one of the following answers
Please choose **only one** of the following:

- Very likely
- Rather likely
- Neutral
- Rather unlikely
- Not likely at all
- No answer

I believe that using a voice assistant would **disrupt the relationship between emergency personnel and patients** during care. *

Choose one of the following answers
Please choose **only one** of the following:

- Strongly agree
- Rather agree
- Neutral
- Rather disagree
- Strongly disagree
- No answer

I believe that using a voice assistant would **assist me in my work** and **reduce my workload**. *

❶ Choose one of the following answers
Please choose **only one** of the following:

- ◯ Strongly agree
- ◯ Rather agree
- ◯ Neutral
- ◯ Rather disagree
- ◯ Strongly disagree
- ◯ No answer

I believe that using a voice assistant would **improve patient care**. *

❶ Choose one of the following answers
Please choose **only one** of the following:

- ◯ Strongly agree
- ◯ Rather agree
- ◯ Neutral
- ◯ Rather disagree
- ◯ Strongly disagree
- ◯ No answer

If you wish, you have the opportunity to leave **additional suggestions or comments** here.

Please write your answer here:

Thank you for your participation!

If you wish, find more detailed information about the project at **www.rescue-research.de** (https://rescue-research.de). Please visit the website again in some time to be informed about the results of this survey (expected from November 2024).

Feel free to leave us your email address: This way we can automatically inform you about the results of this survey. The email address will be stored independently of your survey responses. Click here for that. (https://rescue-research.de/ueber-ergebnisse-informiert-werden/)

Help us make this survey more well-known! Send the survey link to your colleagues or mention it to them. Additionally, you can request posters for display via the website. A higher number of participants means more accurate results for the study. Thank you very much for your support!

09-08-2024 – 23:59

Submit your survey.
Thank you for completing this survey.
