# Supplementary material for: A multinational cross-sectional survey on the use of AI-based voice assistance systems in emergency medical services
Source: Scand J Trauma Resusc Emerg Med. 2026 Sep 15;34:155. doi: 10.1186/s13049-026-01695-1 (PMC13599295; doi:10.1186/s13049-026-01695-1)
Supplement: Supplementary file 2 [file 13049_2026_1695_MOESM2_ESM.pdf]

Möllenhoff, C. et al.: An International Cross-Sectional Survey on Use of AI-based Voice Assistance Systems in Emergency Medical Services

Supplement 2

LLM-Prompt used as foundation for the analysis of multilingual free-text-answers on „Practical Challenges in Deployment“ with ChatGPT Model 5, conducted 14.08.2026

Ich habe in einer Umfrage unter Rettungsdienstmitarbeitern und Notärzten in Deutschland, Norwegen und der Schweiz gemacht. Es geht um die Einführung eines Sprachassistenzsystems im Rettungsdienst. Ich habe ihnen folgende Frage gestellt:

"Sehen Sie Probleme / Herausforderungen, wenn Sie an die Einführung eines Rettungsdienst-Sprachassistenten auf den Fahrzeugen Ihrer Rettungswache denken? Wenn ja, welche?"

Ich schreibe gleich unterhalb als CSV alle Freitextantworten auf diese Frage mit den dazugehörigen Fragebogen-IDs (Spalte "fragebogen-id") und dem Land, aus dem der Teilnehmer der jeweiligen Antwort stammt (Spalte "Land").

Finde Gemeinsamkeiten in den Antworten. Clustere die Antworten auf Themenbereiche, die Aussagen mit ähnlichem Inhalt haben. Eine Aussage kann auch zu mehreren Clustern gehören. Die Anzahl der Cluster kann beliebig groß sein, nicht zu viele und nicht zu wenige. Schreibe bei jedem Cluster dazu, wie viele Antworten du zu diesem Cluster gezählt hast und wie viele jeweils aus welchen Ländern stammen. Schreibe je Cluster alle IDs der Antworten dazu, die du diesem Cluster zugeteilt hast. Gebe je Cluster einen kurzen Text mit dem übergreifenden Inhalt aller Aussagen daraus aus, sodass ich es für eine wissenschaftliche Publikation verwerten kann. Erfinde nichts dazu! Arbeite ausschließlich auf Basis der nun folgenden Eingabe!

fragebogen-id;Land;Antwort

378;ch;"- Falls man Kontraindikationen, Algorithmen oder ähnliches abfragen könnte, geht viel Fachwissen verloren bzw. wird dieses nicht mehr benötigt. Finde ich riskant und schade.

- Man fühlt sich kontrolliert/beobachtet.

- Tonaufnahmen könnten in Notfallsituationen, welche vielleicht negativ verlaufen, gegen jemanden verwendet werden und somit rechtliche Folgen haben. (Beweismaterial)"

207;ch;"- Gesetzgebung

- Kosten"

296;ch;Ablehnung durch ältere Mitarbeitenden

114;ch;Akzeptanz bei älteren Personal

140;ch;"Akzeptanz des Personals und vor allem bei Patienten. Speziell die Älteren - unser Hauptklientel - würden vermutlich AR/VR-Brillen irritierend finden. Auch die Kommunikation mit einer unsichtbaren ""Entität"" im Raum. Sprachausgabe aus dem Nichts ist befremdlich (umgebungslärm) - auch Datenschutz problematisch. Unhörbare Antworten lassen die Frage zusammenhanglos im Raum stehen und es ist unklar wie lange die Antwort dauert bzw. die Fragenden abgelenkt sind."

8;ch;Akzeptanz, konkreter Nutzen über den Gadget-Faktor hinweg, gewisse digitalisierungs-Verdrossenheit da z.B. das digitale Einsatzprotokoll m.E. nicht benuterfreundlich ist. Die Gefahr des Bananen-Prinzips: Das Ding reift beim Kunden (siehe digitales Einsatzprotokoll).

244;ch;Ältere Mitarbeiter

139;ch;Bei Pat. welche nicht akut lebensbedroht sind (Naca 1-3). Beim Transport wird viel geredet. jedoch nicht mit dem Pat., dies könnte zu vielen Missverständnissen führen, und der Pat.-Retter bezug wäre sicherlich sehr speziell.

304;ch;"Crédibilité auprès des patients, introduction du système auprès des personnes réticentes dans le service. En plus, il faudrait une fonction on/Off, car lors d'entretien confidentiel avec un patient, celui ci pourrait de sentir ""écoute"""

174;ch;Datenschutz

384;ch;Datenschutz!

170;ch;Datenschutzbedenken des Betriebs, Kosten, Kooperation der Mitarbeitenden (es muss sofort ein Nutzen spürbar und die Anwendung muss einfach und intuitiv sein).

227;ch;"Datenschutztechnisch

Verlässlichkeit

Anpassungsfähigkeit bei besonderen Wünschen...:)"

108;ch;Die Komplexität und die Schnittstellen für alle Spitäler/Rettungsdienste im föderalistischen System der Schweiz einzuführen. =)

7;ch;Die Wandelsresistenz des Rettungsdienstes insgesamt.

242;ch;"Ein Sprachassistent macht m.E. erst dann Sinn, wenn er tatsächlich die ""normale, freie Sprache"" versteht. D.h. ich muss nicht fixe Formulierungen oder Bausteine verwenden, damit er mich versteht. Dies klappt z.B. bei aktuellen Systemen wie

Alexa oder Siri etc. noch nicht. Gerade im Einsatzfall käme es hier zu Problemen, Anweisungen würden nicht verstanden oder müssten mehrfach wiederholt werden.

Bei verschiedensten Dialekten auf unserer Rettungswache stelle ich mir das Verständnis hier auch eher schwierig vor.

Hier könnte es mittelfristig ggf. mit Hilfe von KI/AI Optimierungen geben."

180;ch;Es braucht immer eine redundante Lösung, damit auch bei einem Ausfall des Systems zuverlässig und in hoher Qualität gearbeitet werden kann.

395;ch;Es gibt viel zu viele Ansichten was dann besser gemacht werden könnte und das dass System nicht von jedem genutzt wird. Wie schwierig oder aufwendig wird die Schulung.

215;ch;Es Muss auch mit Schweizerdeutsch Funktionieren auch mit Dialekt. Das sehe ich als sehr grosse Herausforderung.

150;ch;Es sind nicht alle RD-Mitarbeitenden Digital-Nativs, welche da ggf. mit grossen Herausforderungen konfrontiert werden.

123;ch;Finanzielle Herausforderung und die Herausforderung, jemanden kompetenten zu haben, welcher bei Problemen unterstützen oder Probleme lösen könnte.

326;ch;generational conflict. People who are not comfortable with technology.

179;ch;Gute Schulung. Sollte funktionieren und nicht Fehleranfällig sein.

106;ch;"Haupthürde: die Mitarbeitenden.

Weitere Hürde: Datenschutz-Gesetze (e-Patientendossier in der CH seit Jahren eine ""Baustelle""

Herausforderung: Akzeptanz (Verfügbarkeit) mit diensthabende Ärzte.

Probleme: Finanzierung - Einführung - Daseinsberechtigung gegenüber vielen anderen nicht optimal laufenden Gegebenheiten im CH-Rettungsdienst."

392;ch;"Hohe Kosten.

Implementierungsaufwand von Infos.

Kompatibilität mit den bestehenden Systemen."

240;ch;Ich befürchte am meisten, dass der Sprachassistent nicht unterscheiden kann, ob er oder jemand anders angesprochen wird. Zusätzlich bin ich mir nicht sicher, ob er sowohl in der Ambulanz wie auch im Setting ausserhalb der Ambulanz eingesetzt werden kann.

26;ch;Ich würde dies sehr begrüßen, da es mich schon länger stört, dass ich mit dem eintippen am Tablet viel zu lange habe. Und wenn ich dies gesprochen direkt eingeben könnte, würde es meine Arbeit erleichtern und ich könnte mich mehr auf den Patienten fokussieren.

345;ch;Im Stress Hochdeutsch zu sprechen. Wen Druck da ist, ist es einfacher in der Muttersprache zu sprechen. Daher auch die Sorge, dass die KI Schweizerdeutsch nicht versteht oder nicht 100% richtig verstanden wird und dadurch Fehler passieren. Zudem bedenken, dass das Personal sich zu fest auf die KI verlässt und nicht mehr selbst hinterfragt oder mitdenkt.

91;ch;"In unserem RD bräuchte es für eine entsprechende Umsetzung wohl einiges an Schulung und Aufwand.

Wichtig wäre mir, das der Sprachassistent mich unterstützt, wenn ich das brauche und nicht dazwischenfunkt."

315;ch;Individuelle Bedürfnisse der Mitarbeiter, Kosten, zusätzliche Hardware

133;ch;ja - alle systeme die wir verwenden funktonieren gar nicht oder sehr oft nicht - und falls doch sind alle systeme völlig unbrauchbar - völlig veraltet -sehr sehr langsam - daher kein vertrauten in diesen elektronik schrott - hauptproblem sehe ich darin mich darauf zu verlassen - dann funktoniert doch wieder nichts - mir müssen ein andere lösung suchen - das erzeugt nur stress in heiklen situationen - darum verlasse ich mich auf erfahrung und vorhandenes wissen - ansonst hat mann/frau nur ein medizinisches halbwissen und verlässt sich auf assistenzsysteme - ich sehe darin eine riesige gefahr - anwendung nicht routinemässig sondern wirklich nur bei speziellen situationen -

287;ch;Kann so ein System wirklich verlässlich sein?

241;ch;Kommt schwer auf das Produkt darauf an. Es muss simpel bzw. verständlich und einfach aufgebaut sein. Nicht so fehleranfällig und ziemlich stabil gebaut sein.

17;ch;Kompatibilität mit Umsystemen

229;ch;Kosten

330;ch;"Kosten-/Nutzenfaktor

Datenschutz"

80;ch;Kosten, fehlende Innovationsbereitschaft der Kollegen

100;ch;Kosten, technische Umrüstungen, Benutzerakzeptanz, Zuverlässigkeit/Geschwindigkeit.

334;ch;Les anciens ambulanciers qui seraient réfractaires à la nouvelle technologie

236;ch;Meiner Auffassung nach ist die Affinität für solche Systeme bei den Beteiligten maximal heterogen. Und ja in einigen

Bereichen sind Sprachassistenten sicher von Nutzen hat allerdings aus meiner Sicht nicht die Priorität. So erachte ich es als wichtiger ein digitales Gesamtkonzept zu verfolgen und Fragen wie Zugriff von nichtärztlichen Heilberufen auf elektronische Patientenakten zu ermöglichen Vernetzung der Schnittstellen für aktuell zielführender.

349;ch;Mitarbeiterakzeptanz

286;ch;"Netzabdeckung. Einige Punkte im Einsatzgebiet mit schlechtem beziehungsweise keinem Handyempfang.

Schulung Mitarbeitende. Einbau in bestehende Fahrzeuge"

219;ch;Ob der Sprachassistent mich verseht, oder ob ich z.B. drei mal wiederholen muss, bis er mich versteht (wie z.B. Fernsprechanlage beim Auto, verseht mich ganz schlecht, trotz deutlicher Sprache).

340;ch;Old paramedics that manage EMS since years and that wouldn't be open to this new features.

143;ch;Schulungsaufwand, Akzeptanz

228;ch;"Sich zu sehr auf dieses System verlassen, Wissen geht verloren, denn man kann es ja abfragen.

Umdenken in der Gesellschaft, ev. ist es für den Patienten befremdlich wenn der RS während dem Einsatz mit dem Headset spricht. (Verwirrte oder demente Patienten verstehen nicht dass mit der Technik gesprochen wird)

Spracherkennung in lauter Umgebung möglicherweise schwierig, z.B. bei Bergungen, Strassenrettung, Brand,..."

385;ch;Strukturkonservativismus der Belegschaft („was soll ich damit, ging doch bisher auch ohne")

372;ch;Technische Herausforderung

303;ch;"Technische Voraussetzungen

Verbindung Wlan, Mobilfunknetz etc

Topografische Funklöcher"

257;ch;Trifft auf mich nicht zu.

77;ch;Verlässlichkeit, Betreuung durch Hersteller, Reparaturen und Defekte

184;ch;Verständniss der Beruf

208;ch;We'll have less interactions with patient.

213;ch;Wenn es nicht zuverlässig und schnell ist, wird es im Einsatz nichts nützen

344;ch;Wenn man dialekt redet funktioniert dies nicht und wenn man z.b Mundschutz anhat auch nicht. Wenn es laut ist in der Ambulanz funktioniert dies auch nicht

68;ch;Zu hohe kosten, personal ist nicht offen für veränderungen.

226;ch;"Zusammenarbeit mit diversen verschiedenen Systemen in den Spitälern, Schnittstellen-Problematik

Benutzung darf nie verpflichtend sein

Diverse Mitarbeiter werden versuchen, das System an ihre Vorlieben anzupassen, das darf nicht möglich sein, nur über Systemverantwortliche

Patienten/Angehörige können es als Zeichen von Inkompetenz der Retter empfinden, wenn während des Einsatzes fachliche Anfragen an das System gestellt werden"

293;ch;Zuverlässigkeit der Information/des Systems muss sehr vorgeschritten sein bei der Einfürhung. Ansonsten wird es abgelehnt vom Personal

25;de;"- Kostenfaktor (Kosten-Nutzen)

- Schulungsaufwand

- ggf. gerine Nutzungsrate im Einsatz insb. durch nicht-technikaffine Mitarbeiter

- Datenschutz

- Rückfallebene bei Ausfall der Technik"

59;de;"- Wie nehmen diese moderne Technik ältere Kollegen an?

- Wie wirkt das arbeiten mit diesem System auf die Patienten? Schwindet dadurch das Vertrauen in den Rettungsdienst, die Ausbildung und die Kompetenz?

- Wird wissen von selbst erweitert und neue Leitlinien werden diese noch gelesen oder wird zu 100% auf das Medix vertraut?

- welche auswirkungen hat dieses System dann bei Blackouts oder Katastrophenschutz lagen?"

127;de;"""Haben wir schon immer so gemacht"" Mentalität einiger Kolleg:innen - wenig offen für Neues

Aus Hygiene Gründen personalisierte Headsets sinnvoll= teuer

Headsets sollten mit Hörgeräten kompatibel sein oder das System Bluetooth kompatible Hörgeräte direkt nutzen - wir haben einige Kolleg:innen mit Hörgeräten

System sollte nicht nur im RTW, sondern per Handy/Tablet auch am Einsatzort/ in der Patientenwohnung nutzbar sein, da dort oft 80% der Versorgung passiert"

278;de;"1. Fananzielle: geht nur über verpflichtende Einführung vom Bundesland/Bund und Förderung

2. Personelle (Schulung): insb. bei älteren Kollegen, Teilzeit, Honorarkräften - auch hier sollte eine landesweite/bundesweite Angleichung das Ziel sein.

3. Netzabdeckung: für alle Anwendungen die Internet/Kommunikation mit ILS/Datenbanken etc. erfordern - hier ist bei uns schon das Tele-NA-System an den Grenzen und daher immer noch nicht in den Probegebieten eingeführt."

33;de;Ablehnung durch weniger Technik-BEGABTE Kolleginnen und Kollegen

274;de;"Ablehnung neuer Systeme allgemein.

Ablehnung von Assistenzsystemen aus emotionalen Gründen, „Eingeständnis von Unwissen‟ statt Fokus auf optimale Versorgung

Kosten"

30;de;Akzeptanz bei „Technikmuffeln‟, Finanzierung, hoher Schulungsaufwand

285;de;"Akzeptanz bei Kollegen

Allg. technische Umsetzung

Missbrauch durch dritte Personen

Kommunikationsdefizit mit anderen Einsatzkräften"

115;de;"Akzeptanz der Mitarbeiter

Fehlende Wartung durch den Auftragnehmer

Aktualisierungen der Fachinformationen"

84;de;Akzeptanz der Mitarbeiter, Technik

205;de;"Akzeptanz der Mitarbeiter; Kostenübernahme muss geklärt sein"

177;de;"Akzeptanz des neuen Systems durch Mitarbeiter, unzuverlässigen Spracherkennung bei Eingaben insbesondere in lauten/dynamischen Einsätzen bzw. Hohem Nebengeräuschpegel.

Probleme mit der Verlässlichkeit von KI basierten Übersetzungen."

245;de;Akzeptanz durch Kollegen, Kostenübernahme

50;de;Akzeptanz durch Mitarbeiter

161;de;Akzeptanz durch Mitarbeiter, Patienten, unklarer Funktionsumfang, Ablenkung von wesentlichem

182;de;Akzeptanz wegen fehlender Lernbereitschaft.

190;de;Akzeptanz, Kosten, Verlässlichkeit Kompatibilität

118;de;"Allgemeine Akzeptanz bei den Kollegen; Schnittstellenprobleme mit der Abrechnung, Speicherung der Daten (Volumen, Cloud etc.)"

87;de;"Alte weisse Boomer, die die Dinge ""schon immer so gemacht"" haben. Solche Leute sind Lehr und Lernresistent."

113;de;"Alterspyramide: Je älter die Kollegen, desto schwieriger Akzeptanz zu finden.

Ideales Werkzeug zur Qualiätssicherung: KI kann immer zuhören und aufzeichnen. Auswertung kann vom System anonym erfolgen, insbesondere bei juristischen Fragen von Vorteil (vgl. Dashcam)"

163;de;Am Anfang ist alles schwer, bis es leicht wird...

69;de;Ansprache des Assistenten durch unkooperative Patienten

101;de;Aus Zeitmangel schlecht eingewiesene Mitarbeiter. So als du einen Schulung hattest, wird vorausgesetzt dass man auch alles verstanden hat. Das ist leider neigt immer so und noch viele Fragen bleiben offen.

125;de;Ausfall der Technik und kompliziertere Ersatzlösungen

216;de;"Das missbrauch betrieben wird!

Sprachassistent sollte nur ab drücken Status 4 aktiv werden und sobald Status 1 oder 2 gedrückt ist deaktiviert werden, um nachträgliche Eintragungen und dadurch Fälschung des Einsatzbereiches ausgeschlossen wird!

Digitale Unterschrift durch sagen eines Mitarbeiter spezifischen und selbstgewählten Codeworts"

188;de;Dass es Geld kostet

251;de;Datenschutz. Ablehnende Haltung älterer Mitarbeiter.

58;de;Datenschutz/ Überwachung

28;de;Dauerhafte Internetanbindung nicht gegeben, Ältere Pat. denken, man sei unhöflich

281;de;Dialekte!

276;de;Die Akzeptanz Hilfe oder Unterstützung anzunehmen

249;de;"Die allg. Netzstabilität (WLAN-Verfügbarkeit) in ganz Deutschland.

Geräte die sich daher ständig fest fahren und neu gestartet werden müssen."

95;de;Die Datenschutzrichtlinien in Deutschland

85;de;die deutsche Mentalität

116;de;Die fehlende Compliance mancher Mitarbeiter, die solche Dinge auch im privaten Leben ablehnen und nicht nutzen (wollen).

96;de;"Die Führung würde kein Geld ausgeben. Das muss als gesetzliche Vorgabe gemacht werden/bei der neu Beschaffung bereits integriert sein, dass die Rettungsdienstleitung dies mitmacht.

Datenschutzgrundverordnung.

Mangelnde Fähigkeit in Deutschland Pateineakten auf der Versichertenkarte zu speichern."

341;de;"Die Wachen sind nicht mit WLAN ausgestattet. Updates und Testläufe müssten daher wie auch der Betrieb über SIM-Karten laufen.

Der Mehrwert des Sprachassistenten müsste gesichert sein, die Kostenträger der Refinanzierung zustimmen."

338;de;"Entsprechende Fahrzeuge sind noch nicht vorhanden. Bisher ist es nicht mal möglich das RD Protokoll digital an die Klinik zu senden.

Oft besteht auch keine Verbindung zum Medical Pad Server, mehrfach bereits Ivena Ausfälle."

311;de;Es muss halt verlässlich funktionieren, einigermaßen ausgereift praxistauglich sein (nicht so'n Mist wie NIDA...). Meine Sorge wäre, dass Entwickler ohne Rettungsdienstkenntnisse und Rettungsdienstler ohne IT-Kenntnisse aneinander vorbei reden und man am Ende den Datenschützer gar nicht mehr braucht, weil das Produkt so schon Murks ist.

24;de;Fachliche Kompetenz und Bereitschaft der Annahme der Kollegen

44;de;fehlende Einheitlichkeit des Rettungsdienstes in Deutschland durch föderalistische Strukturen (jeder Kreis macht was er will, jedes Bundesland macht was es will)

255;de;"Fehlende unzureichende Schulung,

Zu viele Fehler bei der Anwendung"

47;de;Finanzielle Umsetzung. Anerkennung der neuen Möglichkeiten von insbesondere älteren Kollegen

259;de;Geld, viel Zeitaufwand zusätzlich zu den Einsätzen, selten wirklicher Bedarf an diesen Systeme, Beherrschung der Systeme auch in seltenen Situationen in denen keine Zeit zum ausprobieren und Testen ist

313;de;Geräuschkontamination und schwierige Umgebungsbedingungen als Störquellen, technische Pitfalls oder/und kompliziertes Handling wird die Mitarbeitercompliance zerstören

117;de;Gewährleistung einer stabilen Internetverbindung, Kompatibilität, Akzeptanz

43;de;"Hacking

Keine Anwendung durch Personal

Missbrauch

Kein sachgemäßer Umgang/keine ordentliche Instandhaltung"

277;de;Hatten wir früher nicht, brauche wir heute nicht (jeder ältere Kollege)

268;de;"Hohes Potential auf Anwenderfehler

Fehlender Innovationsgedanke im Kollegenkreis ("haben wir immer so gemacht")"

388;de;Ich glaube, dass neue Systeme vor allem von älteren Kollegen nur bedingt angenommen werden. Außerdem kann eine verbale Kommunikation mit einem Sprachassistenten im Einsatz zu Verwirrung bei älteren Patienten führen, vor allem wenn diese neurologische vorerkrankt sind oder schlecht hören.

31;de;Ich kann mir bei aller Liebe kein Szenario vorstellen in dem die vorige Schulung in solchen Systemen für alle Mitarbeiter ausreichend umgesetzt wird, aber theoretisch wäre das natürlich schön da so nah wie möglich ranzukommen, denn learning by doing is in manchen Situationen dann doch nicht so toll

122;de;Ich persönlich, nein. Fürchte aber, es gibt reichlich Bedenkenträger zu solchen Systemen.

298;de;"Integration und Bedienung könnte bei älteren, bzw. weniger Tech-affinen Mitarbeitern herausfordernd sein. Zudem könnte Ignoranz ""sowas brauch ich nicht"" und Angst vor Veränderung ""haben wir schon immer so gemacht"" eine Implementierung erschweren"

148;de;Internet auf dem Land, Verständnisprobleme Mensch zu Maschine, Kosten und aufrüsten der Fahrzeuge.

280;de;"Internetanbindung

Integration in bestehende Systeme"

248;de;Ja, die Verlässlichkeit ist nicht gegeben.

185;de;Keine flächendeckende Mobilfunknetzabdeckung

49;de;"Kollegen die sich verweigern es zu nutzen.

Sicherheitsbedenken (kennt das gerät wirklich alle Kontraindikationen, Medikamente)

Versteht mich das system in einer lauten Umgebung (Pressluft horn, FFP2 maske)

Umgang mit Dialekten"

386;de;"Kompatibilität mit RD Hardware (Corpuls, Lifepak, Zoll etc)

Kompatibilität mit Kliniken (müssen Aufträge/Anmeldungen/Rückfragen (Vorbefunde) o.ä. an RD zurückmelden können)

Allgemein Datenschutz

Rechtliche Hürden der Datenübermittlung (in Deutschland glauben in der Medizin immer noch viele, das Fax sicherere als Mail wäre)"

164;de;Kosten/Schnittstellen/Akzeptanz/Eingeitlichkeit

98;de;Kostenaufwand, Schulungsaufwand, Verbauen der Hardware, ggf. viel Bürokratie, fragliche Annahme eines solchen Systems durch die Kollegen

221;de;"Kostenübernahme durch Kostenträger zweifelhaft.

Technische Vorbereitung der Fahrzeuge.

Flächendeckende Verfügbarkeit von adäquatem Mobilfunk."

348;de;MPG Gesetzgebung ist Problematisch gerade bei Hilfestellungen.

3;de;"Neben den potenziellen technischen Schwierigkeiten habe ich die Befürchtung, dass die Akzeptanz gerade bei älteren Kollegen nicht sonderlich hoch sein wird (""sowas haben wir noch nie gebraucht"",etc.). Deswegen ist es vor allem wichtig, alle Mitarbeiter intensiv zu schulen und vor allem auf die Vorteile zu verweisen."

55;de;Netzabdeckung,

375;de;Netzwerkverfügbarkeit (analoge Nutzung Probleme mit Digitalfunk)

358;de;Neutral

196;de;Nutzen der Funktionen, Akzeptanz

306;de;"Oben genannte Probleme bezüglich der Sicherheit und Vertrauenswürdigkeit eines solchen Systems, dürften mehrere Mitarbeiter betreffen.

Zudem dürfte die Funktion des Sprachassistenten auch stark von topografischen Gegebenheiten und dem Ausbau der Infrastruktur wie mobiles Netz und mobiles Internet abhängig sein. In sehr problematischen Regionen wird soetwas vermutlich dann nicht flächendeckend funktionieren, bzw. Sehr störanfällig sein."

38;de;Online Anbindung nicht überall gegeben, Dialektfähigkeit, wenn im Einsatz Störer dazwischen reden - muss das gefiltert werden -

329;de;Patient bemerkt u.U. nicht, dass mit Sprachassistent kommuniziert wird was zu Verwirrung führen kann

45;de;Probleme v.a. im Kontext der Nutzung beim Patienten. Datenschutz-Bedenken der Patienten, Gefühl, dass man als Pat von RD-Personal nicht ernst genommen wird

57;de;Rettungsdienst arbeitet häufig unter akustisch sehr chaotischen Bedingungen. Anfangs nicht so gut entwickelte Systeme könnten die Patientenversorgung verschlechtern

371;de;"Sprachaufnahme und Verständnis bei lauter Umgebung

Unterscheidung der verschiedenen Sprecher (zum Beispiel bei lauten Patienten)

Auf keinen Fall sollten ganze Gespräche dauerhaft aufgezeichnet werden, dennoch sollten vor allem die Antworten des Sprachassistenten dokumentiert werden - was ist wenn man „falsche" Antworten bekommt? Wer haftet?"

9;de;Ständig beschädigte/ zu alte Fahrzeuge

94;de;Störgeräusche, Finanzierung, Fehleranfälligkeit, Unvermögen zum Erkennen von Dialekten

260;de;"stört es vielleicht die Kommunikation mit dem Patienten? diese wird durch Einträge in den Sprachassistenten unterbrochen. man kommuniziert also mit 2 ""Personen"""

142;de;"Überkreuzkommunikation

Kommunikation zwischen RD Personal -Patient ,Kommunikation RD - Personal und als 3. Kommunikation jetzt Sprachassistent,

befürchte zuviel Konversation"

284;de;Umgebungsgeräusche, Akzeptanz

92;de;Verbindung zum Internet in Regionen mit schlechter Verbindung.

310;de;Verkomplizierung und der Versuch, struktuelle Probleme/Unfähigkeit von Kollegen mit noch mehr Technik kompensieren zu wollen anstatt vernüftig und intensiv zu schulen

62;de;Verlässliche Anwendung bei mehreren Personen in RTW, Anwendung in ländlichen Gebieten mit u.U. schlechter Mobilfunkabdeckung

20;de;Verständnisporbleme auf Seiten des Sprachassistenten aufgrund von undeutlicher Sprache oder Störgeräuschen aus dem Einsatzgeschehen

42;de;Vertrauen der Mitarbeiter in das System

282;de;Vielen MA sind nicht (mehr)aufgeschlossen gegenüber neuer bzw zusätzlicher Technik. Alle bisherigen Systeme (IVENA,

NaProt, Telenotarzt sind kaum verknüpft und jeweils separat geschult worden

74;de;Wenig innovationsfreudiges Umfeld

194;de;"Wenig Offenheit für Neues beim Personal,

Sicherheitsbedenken"

218;de;Wenn die Zuverlässigkeit und/oder Bedienbarkeit schlecht ist, wird es zu Widerstand unter den Nutzern kommen.

294;de;Wenn viele gleichzeitig reden, werden schnell Fehler entstehen. Die Unterstützung sollte die Arbeit vereinfachen, nicht komplizierter machen

22;de;Zu hoher Lernaufwand für manche Kollegen („Das hat schon immer so funktioniert."

373;no;Blir vant men ellers ikke noe problemer

343;no;Brukerne

359;no;Dialekt, støy,

337;no;Høye kostnader

382;no;Ja det kan ikke ta for mye fokus fra pasienten, derfor må være enkelt å bruke og fungere bra, om det blir kluss må man kunne overstyre å gjøre ting på «gamlemåten»

351;no;Kan bli en hvilepute når det gjelder egen kunnskap, man trenger ikke huske noe selv og egentlig ikke kunne all verden

356;no;Krav til flygodkjenning. Svært mye støy. Vanskelig å få interaksjon med allerede eksisterende headsett osv

320;no;"Mange eldre/ansatte som har kjørt ambulanse i mange år, som ikke liker endringer.

Ledelse som ikke er interessert i forandring.

Økonomi"

363;no;Mange meninger og derav vanskelig å innføre

357;no;Mer fokus på det teknologiske fremfor pasienten. Følelse for pasient at vi ikke kan det vi skal. Dårlig tilgjengelighet i både bil og hjem.

319;no;"Støy.

Tidsbruk.

Tar fokuset vekk fra pasient, behandling og sikkerhet."

325;no;"Taushetsplikt

Områder med dårlig dekkning

Assistenten burde integreres i nødnett radioen"

370;no;Ulik motivasjon og tilnærming til bruk. Viktig med god opplæring og felles forståelse. Velutprøvde systemer som virker.

365;no;Usikker
