# Supplementary material for: A multinational cross-sectional survey on the use of AI-based voice assistance systems in emergency medical services
Source: Scand J Trauma Resusc Emerg Med. 2026 Sep 15;34:155. doi: 10.1186/s13049-026-01695-1 (PMC13599295; doi:10.1186/s13049-026-01695-1)
Supplement: Supplementary file 3 [file 13049_2026_1695_MOESM3_ESM.pdf]

Möllenhoff, C. et al.: An International Cross-Sectional Survey on Use of AI-based Voice Assistance Systems in Emergency Medical Services

Supplement 3

LLM-Prompt used as foundation for the analysis of multilingual free-text-answers on „Privacy and Data Protection " with ChatGPT Model 5, conducted 14.08.2026

Ich habe in einer Umfrage unter Rettungsdienstmitarbeitern in drei Ländern eine Umfrage gemacht. Es geht um die Einführung eines Sprachassistenzsystems im Rettungsdienst. Ich habe sie gefragt, welche konkreten Bedenken und Sorgen sie zum Thema Datenschutz bei der Einführung und Nutzung eines solchen Systems auf ihrer Arbeit haben. Ich schreibe gleich unterhalb als CSV alle Freitextantworten mit den dazugehörigen Fragebogen-IDs (Spalte "fragebogen-id") und dem Land, aus dem der Teilnehmer der jeweiligen Antwort stammt (Spalte "Land").

Finde Gemeinsamkeiten in den Antworten. Clustere die Antworten auf Themenbereiche, die Aussagen mit ähnlichem Inhalt haben. Eine Aussage kann auch zu mehreren Clustern gehören. Die Anzahl der Cluster kann beliebig groß sein, nicht zu viele und nicht zu wenige. Schreibe bei jedem Cluster dazu, wie viele Antworten du zu diesem Cluster gezählt hast und wie viele jeweils aus welchen Ländern stammen. Schreibe je Cluster alle IDs der Antworten dazu, die du diesem Cluster zugeteilt hast. Gebe je Cluster einen kurzen Text mit dem übergreifenden Inhalt aller Aussagen daraus aus, sodass ich es für eine wissenschaftliche Publikation verwerten kann. Erfinde nichts dazu! Arbeite ausschließlich auf Basis der nun folgenden Eingabe!

fragebogen-id;Land;Teilnehmerantwort

2;de;Ich denke wir übertreiben Datenschutz in Deutschland maßlos und hemmen damit, gerade im Gesundheitswesen Innovationen.

4;de;Gerade, weil ein Med. Notfall immer ein Ausnahmezustand für den Pat. ist, finde ich ein Gerät, das Spracheingabe-basiert arbeitet Datenschutzrechtl. Bedenklich

8;ch;Dass unbeteiligte geschützte Daten mitbekommen, weil ich die ins System spreche oder das System mir diese mitteilt.

9;de;Weitergabe von persönlichen/medizinischen Daten an 3.

17;ch;dass personenbezogene Patientendaten in der Öffentlichkeit landen könnten...

25;de;Laute Aussprache datenschutzrelevanter Informationen

26;ch;Die Abfrage von vorhergehenden Krankenhausaufenthalten denke ich eher als bedenklich

30;de;Sichere Übertragung zwischen verschiedenen Gerätschaften, Auswertung der Daten durch Dritte

31;de;"Komplizierte Kontrolle über Sprachoutput, welche Lautsprecher sind wann wo wie laut, wer hört welche Antworten?

Proprietäre Software ist die Ausgeburt Satans und hat nichts (und ich meine überhaupt garnichts) in kritischer Infrastruktur zu suchen. Das ist geboten aus datenschutzrechtlicher, sicherheitstechnischer und nicht zuletzt auch aus qualitätssichernder Perspektive. Wenn da nicht z.B. ein CCC drübergucken kann und bestätigen kann, dass das nicht kompletter Humbuck ist, dann würde ich das System im Patientensinne nur sehr ungern benutzen. Wenn ihr schon ausschreibt (oder was auch immer, keine Ahnung), dann sollte die Forschung Entwicklung und Umsetzung der Software im Sinne der Allgemeinheit nach freien / öffentlichen Lizenzen erfolgen. Wer das hier auswertet, sollte die zahlreichen Vorteile von FOSS-Software kennen."

33;de;"Mithören durch das ""Umfeld""."

39;de;Speicherung, unbefugte Anhörung, keine sichere Privatsphäre, Schweigepflicht

42;de;Sensible Daten an Unbefugte

44;de;Nicht praxisgerechte Umsetzung, wenn zu aufwendig in der Anwendung wegen Datenschutz (siehe ePA).

45;de;Bei Cloud-basierten Lösungen: Sicherheitslücken in der Übertragung zur Cloud, Angreifbarkeit der Server, …

49;de;"Abzweigen von Pat. Informationen durch nicht befugte dritte. (Datendiebstahl)

Cyperangriffe zur fälschung von daten mit ggf. Patientenschäden"

50;de;Mithören durch Dritte

55;de;Schutz der Daten vor dritten. Beteiligten und unbeteiligten)

57;de;Hackbarkeit und mithören von sensiblen Informationen von dritten (Wiederholung der Informationen)

59;de;- Schutz der persönlichen Daten ggf. in manchen Situation mit umstehenden Passanten oder co. gefährt, jedoch überwiegt für mich die Patienten Sicherheit und das Out-Come dem Datenschutz.

61;de;Mündliche Kommunikation von sensiblen Daten

65;ch;Wenn Informationen zu Drittpersonen gelangen, werde ich dafür rechtlich brlangt? trage ich die Konsequenzen?

80;ch;Aufbewahrung der Daten, Wer greift auf diese Daten zu ?

84;de;Weitergabe von Daten an 3. und kontinuierliche Überwachung und Gesprächsaufzeichnung durch das Gerät

85;de;keine Bedenken solange der Server nicht vom Amazon oder Google ist

91;ch;"Freisprecheinrichtungen sind für JEDEN Zugänglich

Wie unterscheidet der Sprachassistent zwischen einem Befehl von mir oder einer Frage des Patienten?"

92;de;Es muss eine Funktion geben, wo der Sprachassistend sofort aufhört zu reden. Sowas wie: Medix stopp

95;de;Das Mitarbeitende auch an Informationen von neutralen Personen (Keine Patienten) Kommen wollen und diese dann einfach abfragen können.

96;de;Andere können mithören/-sehen. Insbesondere wenn es um sehr persönliche Dinge und Daten geht sehe ich das kritisch(Patientenvorgeschichte weißt einen Schwangerschaftsabbruch auf)

98;de;Eine Anonymisierung der Daten wäre für mich Grundvoraussetzung für ein solches System

100;ch;Die relevanten gesetzlichen Vorgaben (z.B. Datenschutzgesetz) müssen eingehalten werden.

101;de;Zu viele reden, Vorgänge werden angestoßen die nicht sein sollen

103;de;Schlechte technische Implementierung, mithörer

108;ch;Wenn es um Patientendaten geht, wird von Seiten des Datenschutzes oft interveniert. Dies ist eine grosse Hürde.

113;de;Gesunder Menschenverstand beim Umgang mit Daten reicht aus, Datenschutz wird immer auf Kosten der Funktionalität gehen. Wer schwer krank ist interessiert sich nicht für Datenschutz wenn der Rettungsdienst klingelt.

116;de;Das laute Vorlesen von intimen Patienteninfos vor unbefugten Dritten, z.B. Polizei oder Angehörige

117;de;Datenverarbeitung/-speicherung

118;de;"Abhörsicherheit bzw. Fishing der Daten / Informationen; dass es Außenstehende mitbekommen wg- Lautstärke der Sprache"

123;ch;Mithören nicht am Einsatz beteiligter Personen. Abrufen von Daten, welche nicht auf den Einsatz bezogen sind.

127;de;Bei Abfrage von Patientendaten über Lautsprecher könnten Leute außerhalb des RTW zuhören

136;de;Beim Abrufen von Patienteninformationen aus einer digitalen Patientenakte und Ausgabe über Lautsprecher, könnten Leute mithören.

138;de;Gehackt zu werden

139;ch;"Die Daten sind digital Verfügbar, man müsste sicherlich einen gut gesicherten Server betreiben.

Das könnte dazu führen, dass die nachträglicheBeschaffung von Daten mit grossem Aufwand verbunden wäre."

140;ch;Sicherheit der Server bei vielen Schnittstellen. Zuverlässigkeit & Schnelligkeit des Systems/der Verbindung bei strengem Datenschutz

142;de;"Aufgrund der Einsätze oft im öffentlich rechtlichen Raum Zuhörer der ""Schaulustigen"", bei hohem Umgebungslärmlevel muss entsprechend laut gesprochen werden,"

143;ch;Thema Datenschutz ist überbewertet, Sichere Verbindungen sind ein Muss

146;ch;Das sensible Daten missbraucht werden

148;de;Zu viele neugierige Ohren der Bevölkerung

150;ch;es geht um das höchste Gut der Patient:innen, die persönlichsten (medizinischen) Daten, welche auf jeden Fall geschützt werden müssen

158;de;Hochsensible Patientendaten (Personal- sowie Krankheitsdaten) könnten gehackt werden

162;de;Er soll nur auf Anweisung aufzeichnen

163;de;Da wir uns im RD mE in einer art rechtfertigendem Dauernotstand befinden ist der Datenschutzaspekt für Einsatzsituationen vernachlässigbar

167;ch;Patientengeheimnis

168;de;Speichern der Daten, externer Zuvriff

170;ch;Patentenbezogene Daten können nur in Systemen verwendet werden, welche alle gesetzlichen Datenschutzanforderungen erfüllen. Schnittstellen zu anderen (z.B. spitalinternen) Systemen sind auch nur dann möglich. Die Funktionalität und Nutzbarkeit ist also zu einem grossen Teil von einem rigorosen Datenschutz abhängig.

181;ch;Personendaten

182;de;Fehlende Verschlüsselung der Datenübertragung / im Web zugängliche Daten von Patienten.

185;de;Datenablage und Verarbeitung auf Ausländischen Servern mit anderen Sicherheitsstandarts

190;de;Mithören von Bystandern

194;de;Dass Datenschutzvorgaben ein Vorankommen in dem Bereich verhindern

196;de;Online basierte Informationsverarbeitung?

205;de;"Offenlegung von Daten durch Mithörende; Notwendigkeit der Datensicherung auf entsprechenden Servern innerhalb der BRD"

207;ch;dass der Gesetzgeber solche Umsetzungen massiv erschwert

212;ch;Falls es gehakt wird.

215;ch;Ist teils schwierig zum Umsetzten. Nicht Praktisch.

216;de;Das zb die Daten vom Hersteller oder Provider gespeichert werden obwohl dieser sagt und es schriftlich zusichert es nicht zu tun

218;de;Meine größte Sorge: Dass der Datenschutzbeauftragte eine solche Entwicklung verhindert, bevor sie losgehen kann!

220;ch;Es geht um sensible Daten, daher steht hier für mich der Schutz ebendieser an erster Stelle. Ich denke, ein digitaler Sprachassistent kann ein weiterer Schritt Richtung schlechterem Datenschutz sein.

221;de;"Missbrauch der patientenbezogenen Daten im Internet.

Umstehemde Unbeteiligte könnten patientenbezogene Daten irrtümlich mithören."

222;de;Unberechtigter Zugriff

226;ch;"Zugriff auf Patientenakten dürfte sehr schwierig werden, Zustimmung des Patienten/ Passwort des Patienten erforderlich

Standort der Server des Systems müsste unbedingt in Europa sein nicht in den USA"

227;ch;persönliche Daten des Patienten unverschlüsselt zu erhalten und wieder zu versenden finde ich problematisch

228;ch;Plötzliches Verbinden mit Rettungsdienstfremden Geräten und somit auch Dateneinsicht

229;ch;Gerät antwortet so laut, dass unbeteiligte Personen mithören können

231;ch;Augen und Ohren des Umfeldes

236;ch;Also ich würde ungern Firmen wie Google oder Amazon die Möglichkeit bieten einsatzbezogene Informationen mitzuschneiden, bzw. unbefugte Dritte vertrauliche Infos erhalten.

240;ch;"Dass die Daten an Datenbanken weitergeleitet werden, die nichts mit der Behandlung des Patienten zu tun haben, dass diese Daten bei einem Hack ""gestohlen"" werden und für nicht gedachte Zwecke verwendet werden."

241;ch;Mehr Technik - noch weniger Mensch. Ein gesunder Menschenverstand ist immernoch das beste. Diese Tool sollte meiner Meinung nach als Unterstützung genutzt werden. Die Verlockung sich darauf blind zu verlassen ist bei den meisten ziemlich schnell ziemlich gross.

242;ch;Sprachassistenten sind in der Regel Cloud-basiert. Je nach Standort des Servers wäre eine Nutzung datenschutzrechtlich schwierig m.E.

245;de;Hackerangriffe auf Cloudbasierte Systeme. Illegale Abfrage von Daten durch Ermittlungsbehörden.

249;de;Datenschutz verhindert oft die sinnvolle ganzheitliche Nutzung von hilfreichen Lösungen. Beispiel die KK-Gesundheitskarte wird nur für allg. Kontaktdaten des Patienten genutzt.

253;ch;Gleich wie bei jeder digitalen Sammlung von schützenswerten Daten: bevor das System etabliert wird, muss auch ein entsprechender digitaler Schutz gewährleistet sein vor bspw. Hackerangriffen.

255;de;"Gefahr des hacken der Systeme.

Dritte unberechtigt Informationen erhalten bei der Nutzung"

257;ch;"Kompromitierung von Patientendaten.

Monetarisierung der Datennutzung durch Firmen und Konzerne durch Verkauf der erfassten Daten.

Bedenken bei Entscheidern zur Umsetzung eines derartigen Projektes im Kontext von Datenschutz Themen.

Einwilligungsszenarien von betroffenen Patienten und Angehörigen."

259;de;Nachvollziehbarkeit meiner Anfragen durch Arbeitgeber, aussagen des Gerätes sind für alle nachvollziehbar (manchmal soll der Patient die Aussage vielleicht gar nicht wortgetreu hören oder wird durch eine unpräzise Frage verunsichert

260;de;daß wir damit die Arbeit erschweren. wobei dieser Eindruck durch einen Einsatz vor kurzem getriggert wurde: eine Schwester hat mir die Akteneinsicht als behandelnder Arzt mit dem Hinweis auf Datenschutz verweigert

274;de;Führung der Daten über Cloudsysteme in Ländern mit fraglichen Datenschutzregelungen, insb bei Interaktion des KI-Systems mit sonstigen erhobenen Daten (Versicherungsdaten, Digitale Gesundheitsakte, etc)

277;de;Datenverarbeitung, Zuverlässigkeit der Daten

279;de;Manuelle Auswertung (Stichproben) von Anfragen zu Trainingszwecken

281;de;Die Daten dürfen lediglich zur Verbesserung der medizinischen Versorgung verwendet werden. Utilitarismus --> the best for the most

282;de;Zuhörer würden das System in vielen Situationen einschränken oder nicht anwendbar machen.

284;de;Hemmnisse der deutschen Datenschutzbeauftragten

285;de;Die Stimme des Sprachassistent kann von umstehenden Personen gehört werden und dadurch können sensible Daten offengelegt werden. Zusätzlich könnte die Sprachausgabe nicht oder nicht rechtzeitig abgebrochen werden. Auch durch ggf. Missbrauch von dritten Personen welche ebenfalls Sprachbefehle geben und das System verwirren.

286;ch;"Zu viele Schnittstellen. KI wird mit sensiblen Daten (Patientengeschichte etc.) gefüttert, wie sicher ist das? Wie wird die KI vor externem Zugriff geschützt?

Rechtliche Grundlagen erfüllt/nicht erfüllt?"

287;ch;unklar ob die Nutzen wirklich gegeben sind

289;de;Sicherheit vor Hackerangriffen während des Einsatzes.

291;de;Lautsprecher im RTW, die VorErkrankungen offen verkünden

292;de;"Aktuellen gefährden wir durch den Datenschutz unsere Patienten (fehlende Zugriffsmöglichkeit auf Klinikbriefe etc. ...).

Daher habe ich nur Sorgen VOR dem Datenschutz"

293;ch;Schwierige Überprüfbarkeit der Richtigkeit der Information, Speicherung der Speech-Snippets

294;de;Abhörsicher müsste sichergestellt sein. Löschen der Mitschnitte nach Dokumentationsende

296;ch;eher keine Sorgen

303;ch;Datenschutzgesetz in der CH und deren Einhaltung, wenn Pat. Nicht imstande ist seine Zustimmung zu geben.

306;de;"Bezüglich bereits bestehender Sprachassistenten (z.B. Alexa, Siri, usw.) gab es immer wieder Bedenken und Unklarheiten, in Bezug auf die Speicherung und Streuung der ""mitgehörten"" Kommunikation.

Bedeutet, man weiß mithin nie genau wer alles ""zuhört"", wann zugehört wird (auch außerhalb des Einsatzes) , wohin diese Daten gehen und was damit passiert. Gleichwohl ist die Sicherheit,

bzw. Anfälligkeit für Angriffe von außen, eines solches Systems unklar."

310;de;Es sollte eine vernüftige Balance haben

311;de;Dass wirklich gute und hilfreiche Lösungen von den Datenschutzkaspern kaputt diskutiert werden und am Ende wie so oft irgendein halbgarer Mist herauskommt, der die Daten toll schützt, in der Praxis aber nicht zu gebrauchen ist...

313;de;Daten dürfen ausschließlich dem behandelnden Team bzw. dem direkten medizinischen Vorgesetzten (ÄLRD) zugänglich seinp

314;de;Im Fall eines Gerichtsverfahrens

317;ch;Indirekte Informationen an Unbeteiligte

318;ch;Falsche Antworten auf Lautsprecher.

338;de;Sind die Daten gegen dritte von außen geschützt?

341;de;Ich würde den Assistenten nicht für kritische Informationen zum Patienten nutzen und auch eine Bedienung der Medizingeräte durch so einen Assistenten ablehnen. Für Licht an/Licht aus und Wiki-Anfragen zu Leitlinien ist mir der Datenschutz daher nicht wichtig.

342;de;Datensicherheit via Internet, dauerhafte Verarbeitung von Ort und medizinischen Daten, Speicher?

344;ch;Das diese ins Internet geht und jemand es hecken könnte

345;ch;Persönliche Daten des Pat. z.b. Abfragen auf bekannte Vorerkrankungen oder Medikamenten Liste. Woher holt sich die KI diese Information und wie Verlässlich sind diese Informationen?

348;de;Ich habe keine Bedenken, wenn die Sprache nicht vom MA gespeichert werden kann, von der Organisation kann dies geschehen.

349;ch;Systemsicherheit, mobile Datenübertragung

366;de;Ablehnung seitens des Betreibers

371;de;Ich möchte nicht, dass höchst persönliche Daten irgendwo verloren gehen oder außerhalb von Deutschland verarbeitet werden.

372;ch;Wie sicher ist der Datenschutz?

374;de;"Keine Kontrolle, wer auf Daten zugreifen kann.

Durch Audiofunktion können Unberechtigte sensible Daten mithören."

377;de;unbefugte dritte bkeommen zugang zu daten, patienten und angehörige könnten gegen solche systeme aus den o.g. gründen sein

378;ch;Für Drittpersonen oder andere Blaulichtorganisation ungefiltert hörbar, falls System im RTW eingebaut.

384;ch;Das Gerät hört permanent mit. Kann gehackt werden. Kann ggf auch juristisch gegen einem verwendet werden, weil der Einsatz lückenlos dokumentiert wird.

385;ch;Dass überzogene Datenschutzanforderungen oder vorauseilender Gehorsam die Entwicklung eines wirklich hilfreichen Systems behindern oder sogar verunmöglichen

386;de;"Dauerhaftes ""Mithören"" der KI kann uU ausgenutzt und gegen mich verwendet werden. Social Tracking, oder in dem Fall ""med. Tracking"", zb Notarzt XY fragt über KI jedesmal kardiale Notfälle ab... Ist somit nicht fit in dem Bereich. ..Analyse... Verpflichtung zur Schulung oder Anprangerung"

388;de;Ich glaube, dass die DSGVO im Rettungsdienst und auch generell in Deutschland falsch ausgelegt und damit übertrieben angewendet wird. Bedenken habe ich nur gegenüber der Vermittlung der Daten an dritte und dass Patienten schlecht davor ihr Einverständnis geben können.

391;de;Verletzung des Datenschutzes durch lautsprecherbasierte Antworten

392;ch;Allfällige Ersthelfer oder weitere Beteiligte könnten mithören und so an sensible Daten kommen.

395;ch;Das evtl. das gesagte gewertet werden kann ohne dass man ein Bild oder Live dabei war. Somit bietet man einen zusätzliche Angriffsfläche.

13;ch;Zugriff auf die Informationen durch externe Personen oder Medien

208;ch;Jeder

244;ch;Ablage in einer externen Datenbank und somit Zugriff durch Dritte

326;ch;Verlust oder Weitergabe sensibler medizinischer Informationen. Einhaltung des Datenschutzgesetzes und der Geheimhaltungspflicht.

340;ch;Weitergabe sensibler Patientendaten und Verlust des Vertrauens der Patienten.

304;ch;Es handelt sich um sehr sensible Daten und ich möchte nicht, dass meine Daten für jemanden mit bösen Absichten leicht zugänglich sind. Es müsste eine Sicherheitsfunktion geben, damit das System nicht für jedermann zugänglich ist, wie beispielsweise eine PIN auf dem Tablet.

320;no;Erhöhtes Risiko einer möglichen Verletzung der Schweigepflicht

337;no;Außerhalb des Fahrzeugs zu hören.

343;no;Dass das System für Abhörversuche anfällig ist

356;no;Unbefugte können Zugriff auf sehr persönliche Daten erhalten

359;no;Dass andere Personen als der Patient und sein Partner Fragen und Antworten hören

361;no;Personen in der Umgebung können hören, was gesagt wird

363;no;Sich über Patientendaten äußern

365;no;Wenn dies für den Zugriff auf sensible Patientendaten gilt, wo werden diese dann gespeichert? Kann es unter anderem zu Hackerangriffen kommen?

370;no;Keine Bedenken

373;no;Keine. Vertrauen in das System und die Vorschriften

393;no;Wenn Angehörige oder andere Personen sensible Patientendaten hören können
