# Supplementary material for: A multinational cross-sectional survey on the use of AI-based voice assistance systems in emergency medical services
Source: Scand J Trauma Resusc Emerg Med. 2026 Sep 15;34:155. doi: 10.1186/s13049-026-01695-1 (PMC13599295; doi:10.1186/s13049-026-01695-1)
Supplement: Supplementary file 4 [file 13049_2026_1695_MOESM4_ESM.pdf]

Möllenhoff, C. et al.: An International Cross-Sectional Survey on Use of AI-based Voice Assistance Systems in Emergency Medical Services

Supplement 4

Equation 1 and Equation 2: Calculation of scores for Non-Functional Requirements

Equation 1	$s_m = \sum_{p=0}^{M-1} x_{m,p} \times (M - p)$
Equation 2	$g_m = \frac{s_m}{\sum_{i=1}^{M} s_i}$

Legend Equation 1 and Equation 2: M = Amount of all NFR (here=8), m = individual NFR (m ∈M), s_m = score of individual NFR, g_m = weight of the individual NFR among all NFR, p = priority (0…7,whereas 0 highest), $x_{(m,p)}$ = number of participants who rated NFR m with priority p
